# A Noninvasive Tool Based on Magnetic Resonance Imaging Radiomics for the Preoperative Prediction of Pathological Complete Response to Neoadjuvant Chemotherapy in Breast Cancer

**DOI:** 10.1245/s10434-022-12034-w

**Published:** 2022-06-30

**Authors:** Chenchen Li, Nian Lu, Zifan He, Yujie Tan, Yajing Liu, Yongjian Chen, Zhuo Wu, Jingwen Liu, Wei Ren, Luhui Mao, Yunfang Yu, Chuanmiao Xie, Herui Yao

**Affiliations:** 1 Guangdong Provincial Key Laboratory of Malignant Tumor Epigenetics and Gene Regulation, Department of Medical Oncology, Breast Tumor Centre, Phase I Clinical Trial Centre, Sun Yat-sen Memorial Hospital, Sun Yat-sen University, Guangzhou, China; 2 Department of Nasopharyngeal Carcinoma, State Key Laboratory of Oncology in South China, Collaborative Innovation Center for Cancer Medicine, Guangdong Key Laboratory of Nasopharyngeal Carcinoma Diagnosis and Therapy, Sun Yat-sen University Cancer Center, Guangzhou, Guangdong China; 3 Department of Medical Oncology, The Third Affiliated Hospital of Sun Yat-sen University, Guangzhou, China; 4 Department of Radiology, Sun Yat-sen Memorial Hospital, Sun Yat-sen University, Guangzhou, China; 5 Division of Science and Technology, Beijing Normal University-Hong Kong Baptist University United International College, Hong Kong Baptist University, Zhuhai, China; 6 Department of Medical Imaging, State Key Laboratory of Oncology in South China, Collaborative Innovation Center for Cancer Medicine, Guangdong Key Laboratory of Nasopharyngeal Carcinoma Diagnosis and Therapy, Sun Yat-sen University Cancer Center, Guangzhou, China

## Abstract

**Purpose:**

This study aimed to identify patients with pathological complete response (pCR) and make better clinical decisions by constructing a preoperative predictive model based on tumoral and peritumoral volumes of multiparametric magnetic resonance imaging (MRI) obtained before neoadjuvant chemotherapy (NAC).

**Methods:**

This study investigated MRI before NAC in 448 patients with nonmetastatic invasive ductal breast cancer (Sun Yat-sen Memorial Hospital, Sun Yat-sen University, *n* = 362, training cohort; and Sun Yat-sen University Cancer Center, *n* = 86, validation cohort). The tumoral and peritumoral volumes of interest (VOIs) were segmented and MRI features were extracted. The radiomic features were filtered via a random forest algorithm, and a supporting vector machine was used for modeling. The receiver operator characteristic curve and area under the curve (AUC) were calculated to assess the performance of the radiomics-based classifiers.

**Results:**

For each MRI sequence, a total of 863 radiomic features were extracted and the top 30 features were selected for model construction. The radiomic classifiers of tumoral VOI and peritumoral VOI were both promising for predicting pCR, with AUCs of 0.96 and 0.97 in the training cohort and 0.89 and 0.78 in the validation cohort, respectively. The tumoral + peritumoral VOI radiomic model could further improve the predictive accuracy, with AUCs of 0.98 and 0.92 in the training and validation cohorts.

**Conclusions:**

The tumoral and peritumoral multiparametric MRI radiomics model can promisingly predict pCR in breast cancer using MRI images before surgery. Our results highlighted the potential value of the tumoral and peritumoral radiomic model in cancer management.

**Supplementary Information:**

The online version contains supplementary material available at 10.1245/s10434-022-12034-w.

Breast cancer is the most common carcinoma in women worldwide, with more than 90% of patients with breast cancer diagnosed in stages I–III.^1,2^ Neoadjuvant chemotherapy (NAC) is recommended to reduce tumor burden and eventually reach radical surgical interventions.^3^ As the ideal outcome of NAC, pathological complete response (pCR) in both the primary lesion and lymph nodes (ypT0/is, ypN0) is associated with less aggressive surgery and better overall survival (OS).^4^ Previous studies proposed that pCR could be used as a substitute short-term endpoint for long-term survival in patients with breast cancer.^5,6^ However, approximately 50–70% of patients receiving NAC failed to achieve pCR.^7,8^ Therefore, predicting pCR before NAC is urgently needed for the treatment decision of breast cancer patients.

Imaging examinations, such as ultrasound, mammography, and magnetic resonance imaging (MRI), are routinely used for characterization of breast cancer. Among these techniques, MRI is the most reliable method for identifying pCR before NAC.^9–11^ However, a meta-analysis study indicated that the sensitivity of MRI for pCR was highly variable, with a pooled sensitivity of only 64%.^12^ Recently, radiomics involving high-throughput extraction of computational imaging features have emerged as a promising strategy for breast cancer evaluation.^13–16^ By means of radiomics, we can capture and analyze the high-dimensional image data to allow an in-depth characterization of tumor in a noninvasive manner. Previous research demonstrated that multiparametric MRI-based radiomics is promising and reliable to determine the response to NAC in breast cancer patients.^17–22^

However, the previous prediction models for pCR mostly focused on tumor region. Emerging evidence suggested that the heterogeneity of tumor microenvironment (TME) might contribute to the development, progression, and metastasis of cancer.^23^ In recent studies, radiomic models for imaging of the peritumoral region showed favorable performance in molecular subtyping,^13^ therapeutic response monitoring,^24^ and risk stratification^25^ for multiple malignant tumors. Additionally, recent studies have demonstrated the promising prediction value of peritumoral MRI in NAC response prediction for breast cancer.^26^ The peritumoral MRI may provide additional peritumoral microenvironment information on predicting pCR in breast cancer patients.

The present study focused on both the tumoral and peritumoral regions of pretreatment MRI. We aimed to construct and validate a machine learning-based radiomic model to predict pCR to NAC before surgery in patients with breast cancer.

## Methods

### Patients and Study Design

In this retrospective study, we initially recruited 657 patients, from two institutions, with nonmetastasis invasive breast cancer who received NAC as planned before surgery. The inclusion criteria were (1) patients pathologically diagnosed with primary invasive breast cancer; (2) radical surgery performed after completion of NAC; and (3) breast MR examination conducted within 2 weeks before NAC. Radical surgery refers to the resection of primary tumor lesion with negative margin. The specific surgical methods include segmental mastectomy breast-conserving surgery, nipple-sparing mastectomy, simple mastectomy, modified radical mastectomy, and radical mastectomy for breast cancer. The exclusion criteria were (1) patients with nonductal breast cancer; (2) MRI images insufficient to assess efficacy; (3) treatment other than NAC had been performed before the operation; and (4) patients with unavailable postoperative pathologic results or pCR was not assessed. Of these, 448 patients met the criteria for the final analysis.

For the training cohort, 362 patients treated at the Sun Yat-sen Memorial Hospital, Sun Yat-sen University (SYSMH, Guangzhou, China) between 17 November 2011 and 25 December 2018 were enrolled. Another 86 patients treated at the Sun Yat-sen University Cancer Center (SYSUCC, Guangzhou, China) between 26 February 2015 and 23 July 2019 were enrolled in the validation cohort.

NAC was performed according to the standard protocol in National Comprehensive Cancer Network (NCCN) guidelines for breast cancer.^27^ Patients received at least four cycles of treatment according to the response to NAC. The regimens could be anthracycline-based, taxane-based, or anthracycline and taxane-based. Additionally, human epidermal growth factor receptor 2 (HER2)-positive patients received chemotherapy combined with trastuzumab (8 mg/kg as the loading dose and 6 mg/kg as the maintenance dose). The Ethics Committee of all included institutions approved this retrospective analysis of anonymous patient data.

### Outcomes

The primary endpoint of this study was pCR, defined as no invasive residual cancer cells in either breast or axillary lymph nodes after NAC (ypT0/is, ypN0). Postoperative pathologic staging was evaluated by experienced pathologists via the standard histopathologic analysis of complete resected surgical specimens and all nearby regional lymph node specimens.

### Magnetic Resonance (MR) Image Acquisition

All patients underwent an MRI examination before biopsy but within 2 weeks before NAC. Axial MRI images of the bilateral breast were obtained. This study selected three imaging sequences, i.e. contrast-enhanced T1-weighted imaging (T1+C), T2-weighted fat suppression imaging (T2WI), and diffusion-weighted imaging quantitatively measured the apparent diffusion coefficients map (DWI-ADC) for further analysis. Detailed information on MRI scanning parameters is provided in electronic supplementary Table S1.

### Volume of Interest Masking and MR Feature Extraction

The MRI images of all qualified patients were collected for volumes of interest (VOI) segmentation and MR image features extraction. The VOIs were manually drawn via commercial research software (3D Slicer 4.10.2, https://www.slicer.org).

Since the MRI examinations were performed using different MR equipment in the two institutions, all images were pretreated with the N4ITK MR bias correction module before segmenting in order to standardize the distribution of the magnetic field of all samples. After homogenization pretreatment, the Segment Editor module was applied to manually delineate VOI segmentations on each tumor or peritumor layer of the three selected sequences. The VOI images (DICOM format) were then transferred to the Slicer Radiomics code. The closed surface of VOIs could be visualized in the three-dimensional (3D) view. For each patient, we delineated two separate masks as follows: tumor lesion VOI (tumoral VOI) and peritumoral region, which contained a 10-mm area surrounding the tumor lesion VOI (peritumoral VOI). The 3D VOIs were semi-automatically segmented by two radiologists and two oncologists (CL, NL, ZH, and YY), and were further evaluated by two senior radiologists (ZW and CX) with more than 15 years’ experience in breast MRI interpretation. ZW and CX were chiefly responsible for evaluation of the VOIs. The Breast, Imaging, Reporting and Data System (BI-RADs) standards were used to define the boundary of the VOIs.^28^ Any disagreements were resolved by consensus after consultation between the radiologists and the corresponding author. The radiomics extension module^29,30^ was used to extract MR imaging features. A total of 863 quantitative features of each imaging sequence were extracted from each VOI of each patient (electronic supplementary Table S2). The radiomics included 12 diagnostic features, 107 original features (first-order, shape, gray-level co-occurrence matrix [GLCM], gray-level size zone matrix [GLSZM], gray-level dependence matrix [GLDM], and neighborhood gray-tone difference matrix [NGTDM] features), and 744 wavelet features. The final feature set incorporated 2589 features for each VOI, and a total of 5178 features were extracted from each patient. The extracted radiomic feature intensities were then normalized to adjust values to a notionally common scale of between −1 and 1.

### Prediction Model Construction

First, the Mann–Whitney *U* test was used to select the features with statistical significance associated with pCR or non-pCR prediction (*p* < 0.05), and second, the random forest (RF) was used to sort the top 20 and top 30 key features of each MRI sequence in two VOIs for pCR prediction. Finally, supporting vector machine (SVM) models were used to construct a prediction model with the top 20 and top 30 key radiomic features for pCR prediction, respectively. The tumor VOI multiparametric MRI radiomics model was constructed based on a combination of the T1+C, T2WI, and DWI-ADC sequences. Analogously, the peritumoral VOI multiparametric MRI radiomics model and tumor + peri VOI multiparametric MRI radiomics model were also constructed based on the above three sequences. The receiver operating characteristic (ROC) curve and area under the curve (AUC) were calculated to assess the predictive accuracy of the radiomics classifier.

### Clinical Prediction Model Construction

First, the univariate and multivariate analyses were used to select the significant clinical factors for pCR prediction, and the selected factors were then used for model construction via an SVM algorithm. The ROC curves and AUCs were calculated to assess the predictive accuracy of the clinical prediction model.

### Statistical Analysis

Descriptive data were expressed in terms of frequency and percentage. Continuous parametric variables were shown as means ± standard deviation (SD) and nonparametric variables were shown as median (interquartile range [IQR]). The comparison of categorical variables was performed using the Pearson Chi-square test or Fisher’s exact test, while continuous variables were tested using the Mann–Whitney *U* test or Student’s *t* test. All statistical tests were two­sided and *p*-values <0.05 indicated a statistically significant difference. All statistical analyses were performed using R software (http://www.R-project.org) and SPSS 24.0 (IBM Corporation, Armonk, NY, USA).

## Results

### Baseline Characteristics

**Fig. 1:**
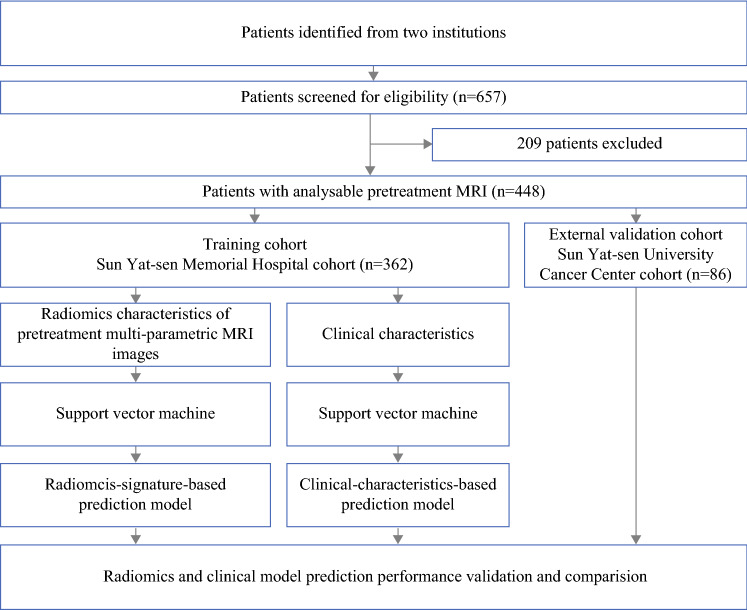
Patient recruitment and study design. *MRI* magnetic resonance imaging

A total of 448 patients with primary invasive ductal breast cancer were finally enrolled (Fig. 1). The median age was 46 years (IQR 39–53) and the median tumor size was 3.6 cm (range 0.6–12.0 cm). Among all patients, 55 (12.1%) patients reached pCR, while 399 (87.9%) were placed in the non-pCR group. The pCR rate in the training and validation cohorts was 10.5% and 18.5%, respectively. Patients with estrogen receptor- and/or progesterone receptor-positive and HER2-negative status were placed in the hormone receptor (HR)-positive subgroup, accounting for 52.2% of patients; patients with HER2-positive status were placed in the HER2-positive subgroup, accounting for 39.7% of patients; and patients with HR-negative and HER2-negative status were placed in the triple-negative subgroup, accounting for 8.1% of patients. For TNM stage, 56% patients had stage I–II disease and 44% had stage III disease. Demographic and clinical baselines of the training and validation cohorts are summarized in Table 1.

**Table 1:** Clinical and histologic characteristics of the entire, training, and validation cohorts

Characteristics	Entire cohort [*N* = 448]	Training cohort [*n *= 362]	Validation cohort [*n *= 86]	*p-*Value
Age, years [median (IQR)]	46 (39–53)	46 (40–53)	48 (39–54.8)	0.5822
No. of tumors				< 0.0001
1	364 (81.2)	311 (85.9)	53 (61.6)	
>1	84 (18.8)	51 (14.1)	33 (38.4)	
Clinical *T* stage				< 0.0001
*T*1	18 (4.0)	8 (2.2)	10 (11.6)	
*T*2	251 (56.0)	212 (58.6)	39 (45.3)	
*T*3	132 (29.5)	113 (31.2)	19 (22.1)	
*T*4	47 (10.5)	29 (8.0)	18 (20.9)	
Clinical *N* stage				0.1582
Negative	66 (14.7)	58 (16.0)	8 (9.3)	
Positive	382 (85.3)	304 (84.0)	78 (90.7)	
Clinical TNM stage				0.0004
I–II	251 (56.0)	218 (60.2)	33 (38.4)	
III	197 (44.0)	144 (39.8)	53 (61.6)	
ER status				<0.0001
Negative	85 (19.0)	51 (14.1)	34 (39.5)	
Positive	363 (81.0)	311 (85.9)	52 (60.5)	
PR status				0.6393
Negative	175 (39.1)	139 (38.4)	36 (41.9)	
Positive	273 (60.9)	223 (61.6)	50 (58.1)	
HER2 status				0.0113
Negative	270 (60.3)	229 (63.3)	41 (47.7)	
Positive	178 (39.7)	133 (36.7)	45 (52.3)	
Ki67 status				0.2923
< 14	28 (6.2)	20 (5.5)	8 (9.3)	
≥ 14	420 (93.8)	342 (94.5)	78 (90.7)	
Molecular subtypes				< 0.0001
HR-positive	234 (52.2)	202 (55.8)	32 (37.2)	
HER2-positive	178 (39.7)	133 (36.7)	45 (52.3)	
Triple negative	36 (8.1)	27 (7.5)	9 (10.5)	

Data are expressed as *n* (%) unless otherwise specified

*IQR* interquartile range, *T* tumor, *N* node, *TNM* tumor-node-metastasis, *ER* estrogen receptor, *PR* progesterone receptors, *HER2* human epidermal growth factor receptor 2, *Ki67* proliferation marker protein Ki-67, *N or n* no. of patients

### Radiomic Models for Pathological Complete Response Prediction

**Fig. 2:**
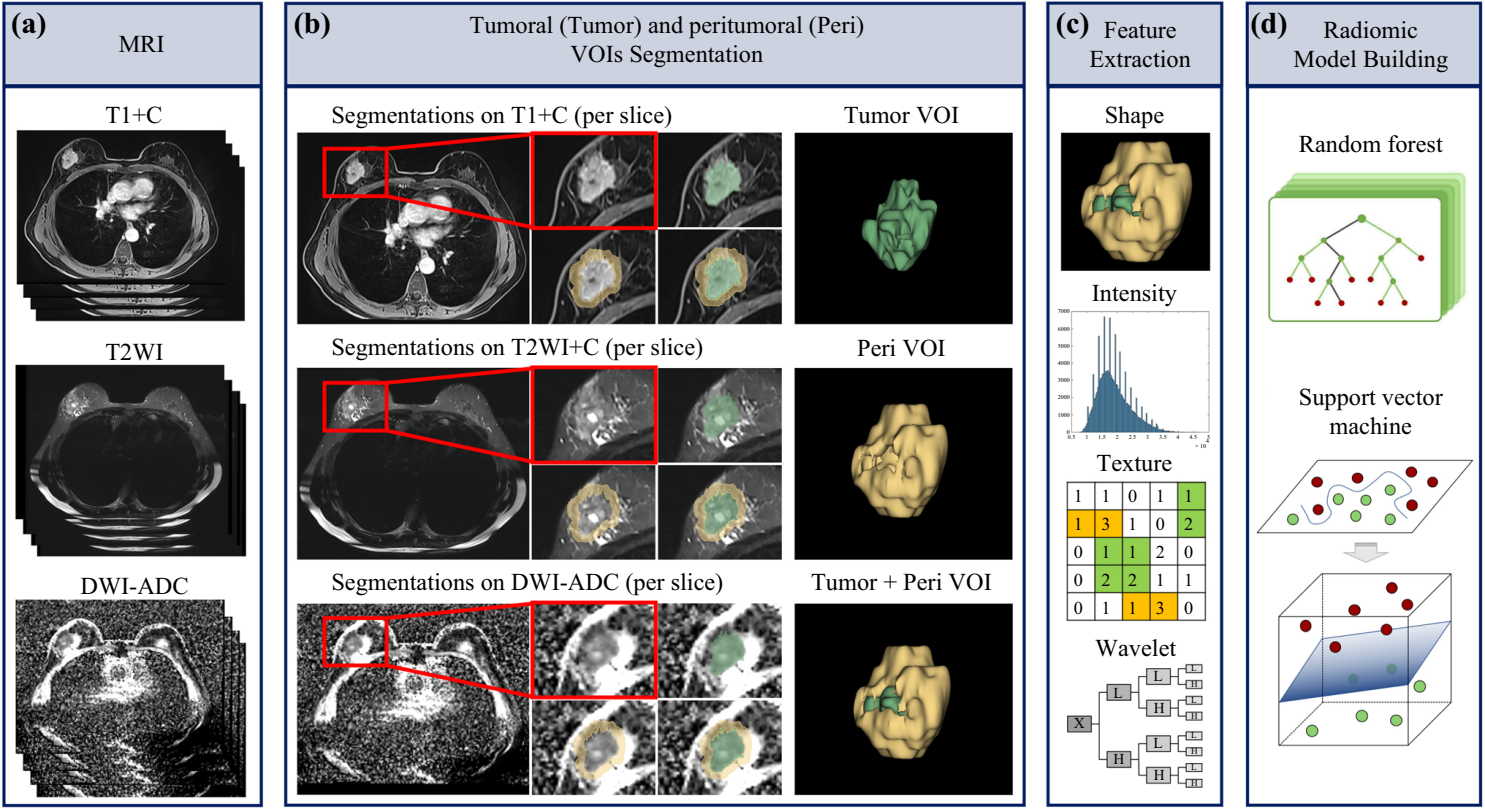
Radiomics workflow. **a** T1+C, T2WI, and DWI-ADC sequences of multiparametric MRI images were recruited for analysis. **b** Tumoral and peritumoral ROIs were identified and segmented slice by slice on T1+C sequence, and ROIs were respectively synthesized into 3D VOIs automatically using software. VOIs were copied to two other sequences for the next analysis. **c** The features of each sequence and each VOI were extracted using 3D Slicer software, including shape, intensity, texture, and wavelet features. **d** Random forest algorithm and support vector machine methods were mainly used for key features selection and radiomic model construction. *T1+C* contrast-enhanced T1-weighted imaging, *T2WI* T2-weighted imaging, *DWI-ADC* diffusion-weighted imaging quantitatively measured the apparent diffusion coefficient, *MRI* magnetic resonance imaging, *ROI* region of interest, *VOI* volume of interest, *3D* three-dimensional

A total of 5178 radiomic features from the T1+C, T2WI, and DWI-ADC sequences of both the tumoral VOI and peritumoral VOI of each patient’s MRI were extracted and analyzed, as shown in Fig. 2 and listed in electronic supplementary Table S1. Using the Mann–Whitney *U* test, we coarsely selected imaging features with a *p*-value <0.05 between pCR and non-pCR patients. The RF algorithm was used to rank the above selected features and the top 30 features of each sequence were selected. The top 20 and top 30 key radiomic features were used for further single-sequence SVM model construction. The optimal number of features was determined as top 30 for better performance (electronic supplementary Table S3). The classification of these features is shown in electronic supplementary Table S4.

**Fig. 3:**
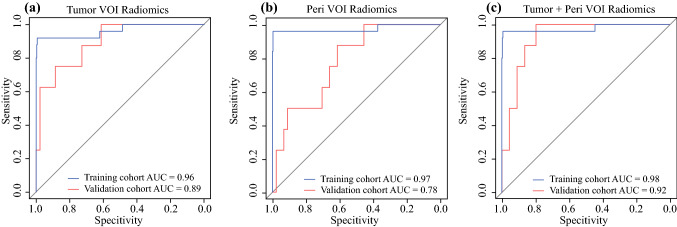
Performance of the three radiomic signatures in the training and validation cohorts. **a** AUC of the tumor VOI multiparametric MRI radiomics model, **b** the peri VOI multiparametric MRI radiomics model, and **c** the tumor + peri VOI multiparametric MRI radiomics model in the training and validation cohorts. *AUC* area under the curve, *tumor VOI* tumoral volume of interest, *peri VOI*, peritumoral volume of interest, tumor + peri VOI, tumoral and peritumoral volumes of interest, *MRI* magnetic resonance imaging

**Fig. 4:**
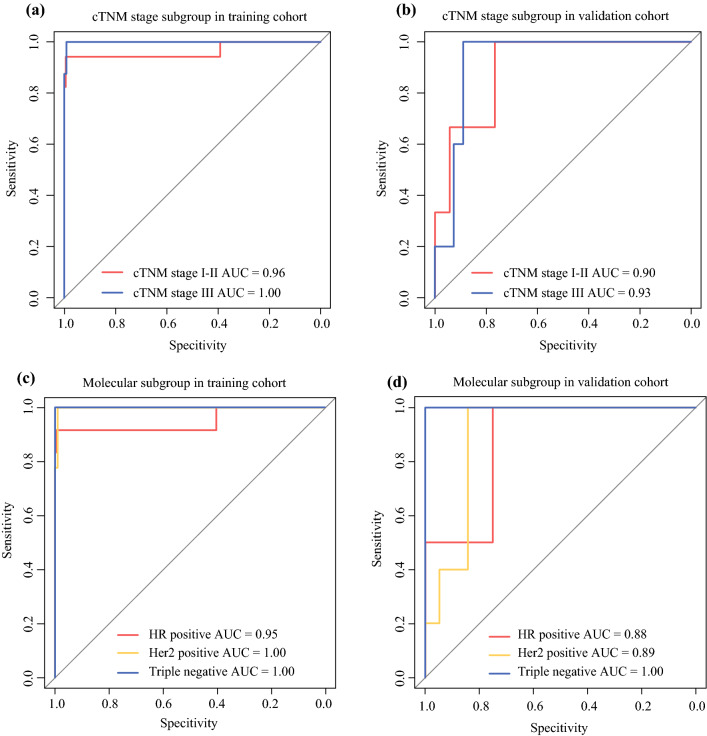
Subgroup analysis of the tumoral + peritumoral multiparametric MRI radiomics model. AUC of subgroup analysis stratified by cTNM stage in the **a** training and **b** validation cohorts. AUC of subgroup analysis stratified by molecular subgroup in the **c** training and **d** validation cohorts. *MRI* magnetic resonance imaging, *AUC* area under the curve, *cTNM stage* clinical tumor, node, and metastasis stage, *HR* hormone receptor, *HER2* human epidermal growth factor receptor 2

The ROC curves and AUCs of the multiparametric MRI radiomics models in the training and validation cohorts are shown in Fig. 3. Not surprisingly, the multiparametric MRI radiomics model of the tumoral or peritumoral VOIs demonstrated better performance than all single-sequence radiomic models in both cohorts. The AUCs of the combined-sequence tumor VOI model were 0.96 and 0.89 in the training and validation cohorts, respectively, and the AUCs of the combined-sequence peritumoral VOI model were 0.97 and 0.78 in the training and validation cohorts, respectively. The combined-sequence radiomic model of the tumor + peritumoral VOI showed the highest predictive accuracy, with AUCs of 0.98 and 0.92 in the training and validation cohorts, respectively. More detailed results are summarized in electronic supplementary Fig. S1 and Table S5. In addition, subgroup analysis indicated that a multiparametric MRI radiomics model of tumor + peritumoral VOI also performed well in different clinical stages, molecular subgroups, and other subgroups with AUCs ≥0.90 (Fig. 4, and electronic supplementary Fig. S2, Table S6, and Table S7).

### Clinical Model for Pathological Complete Response Prediction

We further performed logistic regression analyses of clinical characteristics related to the pCR status of patients, and the results are summarized in electronic supplementary Table S8. In both cohorts, pCR was significantly associated with clinical TNM stage and molecular subgroups. The clinical model was then constructed with an AUC of 0.54 and 0.81 in the training and validation cohorts, respectively. The AUCs indicated that the predictive accuracy of the multiparametric MRI radiomics model was superior to the clinical model in both the training and validation cohorts (electronic supplementary Fig. S3).

## Discussion

In this multicenter study, we constructed a high performance model incorporating tumoral and peritumoral radiomic models of preoperative MRI for pCR prediction before NAC in patients with nonmetastatic invasive ductal breast cancer. Our machine learning-based radiomics might provide a noninvasive tool for individualized pCR prediction and help clinicians with the treatment strategy decision for patients with breast cancer. This study was novel in investigating radiomics incorporating tumor lesions and peritumor regions for predicting response to NAC in breast cancer.

Evaluation of the response to NAC for patients with breast cancer using conventional clinicopathological characteristics remains a challenge for clinicians. Previous studies demonstrated that radiomic features could be a manifestation of the intrinsic identities of tumor lesions and were thus valuable for NAC response prediction.^11,17,18,31–33^ Liu et al.^18^ recently developed an SVM model using multiparametric MRI-based radiomic features of tumor lesions, which resulted in an AUC of 0.68–0.79 for discriminating pCR in patients with breast cancer. Despite multiple radiomic signatures being developed to predict the response to NAC, most of them only focus on the tumor lesion regions.

Recent studies demonstrated that the TME could play a key role in the response to NAC in several solid tumors.^13,34^ Although radiomics have been widely used for the full-course management of malignant tumors, a paucity of models that focus on the peritumoral volumes exists. Hu et al. suggested that the combination of peritumoral signatures can improve the discriminatory power of the computed tomography-based radiomic model, with an AUC >0.85 for pCR prediction in patients with esophageal squamous cell carcinoma receiving neoadjuvant chemoradiation.^35^ Furthermore, Xu et al. demonstrated that combining tumoral and peritumoral radiomic signatures can achieve higher accuracy in the prediction of microvascular invasion and outcome in hepatocellular carcinoma.^36^ In an attempt to comprehensively develop a robust MRI-based radiomics prediction model for promising clinical application, we incorporated the radiomic features of tumoral and peritumoral VOIs to discriminate pCR patients before treatment.

Our findings indicate that the radiomic signature of tumoral and peritumoral VOIs was promising for predicting pCR, with AUCs of 0.89 and 0.78 in validation cohort, respectively. Moreover, model performance could be further improved using the tumoral + peritumoral VOI combined-sequence radiomics signature. Despite differences in the MRI acquisition parameters among different medical centers, our tumoral + peritumoral VOI radiomics demonstrated robust performance with an AUC of 0.92 in the validation cohort. Indeed, our results confirmed that peritumoral radiomics might provide an additional value in treatment response prediction, and multi-VOI integration achieved superior prediction performance compared with a single VOI.

Our study was subject to several limitations. First, this was a retrospective study and selection bias was inevitable. Admittedly, the pCR rate in our study was lower than the present ideal level, which might partly result from the high baseline tumor load and wide range of timespan for our cohorts. The treatment strategies and NAC response have improved greatly in recent years. Second, some baseline clinicopathologic differences were observed between the two institutions, for more patients with advanced tumors seeking care from the cancer center (SYSUCC). Despite the selection bias, our radiomic models showed good predictive performance in both cohorts, suggesting the great generalizability of the models to the real-world population. Third, the tumor and peritumor segmentation of the VOI was performed in a manually automated manner, which was time-consuming and relied on domain expertise. Artificial intelligence methods that perform precise automatic segmentation are needed for further clinical application. In addition, all the enrolled cases in this study were confined to the Chinese population and the effect of ethnic differences on the outcome for patients with breast cancer was not included.

## Conclusion

Our study presented an MRI-based machine learning model combining tumoral and peritumoral radiomics that accurately predicted pCR before surgery. Our results highlighted the potential clinical value of tumor and peritumoral region radiomics-based feature classifiers to predict pCR to NAC.

## Supplementary Information

Below is the link to the electronic supplementary material.

Supplementary file1 (DOCX 475 KB)
