# Supplementary material for: A Noninvasive Tool Based on Magnetic Resonance Imaging Radiomics for the Preoperative Prediction of Pathological Complete Response to Neoadjuvant Chemotherapy in Breast Cancer
Source: Ann Surg Oncol. 2022 Jun 30;29(12):7685–93. doi: 10.1245/s10434-022-12034-w (PMC9550709; doi:10.1245/s10434-022-12034-w)
Supplement: Supplementary file 1 — Supplementary file1 (DOCX 475 KB) [file 10434_2022_12034_MOESM1_ESM.docx]

**Figure S1. Performance of single-sequence radiomic models**


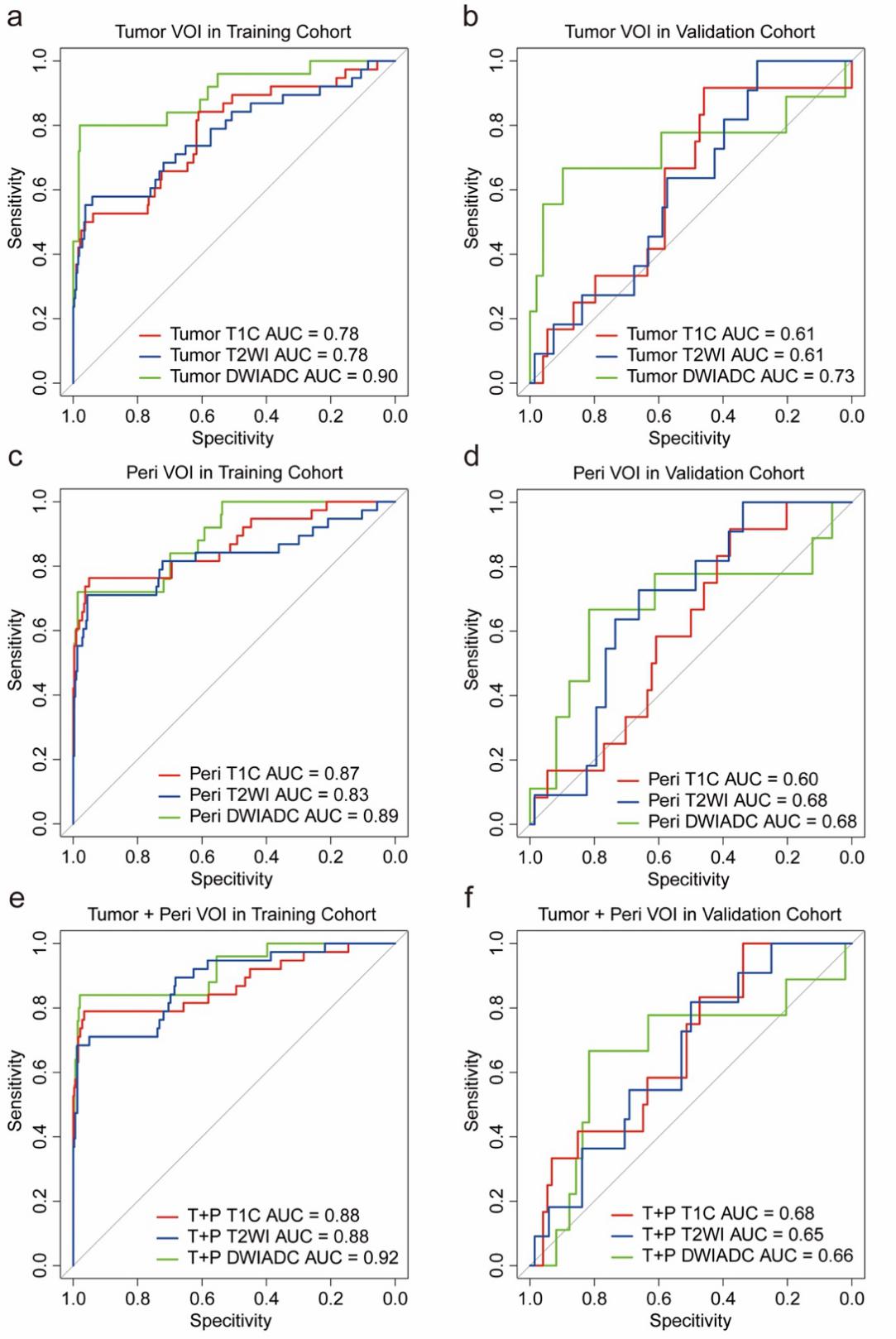


AUC of the Tumor VOI single-sequence radiomic model (A-B), the Peri VOI single-sequence radiomic model(C-D), and the Tumor + Peri VOI single-sequence radiomic model (E-F) in the training and validation cohorts. AUC, area under curve; MRI, magnetic resonance imaging; T1+C, contrast-enhanced T1-weighted imaging; T2WI, T2-weighted imaging; DWI-ADC, diffusion-weighted imaging quantitatively measured the apparent diffusion coefficient; VOI, volume of interest; Tumor VOI, tumoral volume of interest; Peri VOI, peritumoral volume of interest; Tumor + Peri VOI, tumoral and peritumoral volume of interest; T + P, tumoral and peritumoral.

**Figure S2. Performance of tumoral + peritumoral multiparametric MRI radiomics model in subgroup analysis**


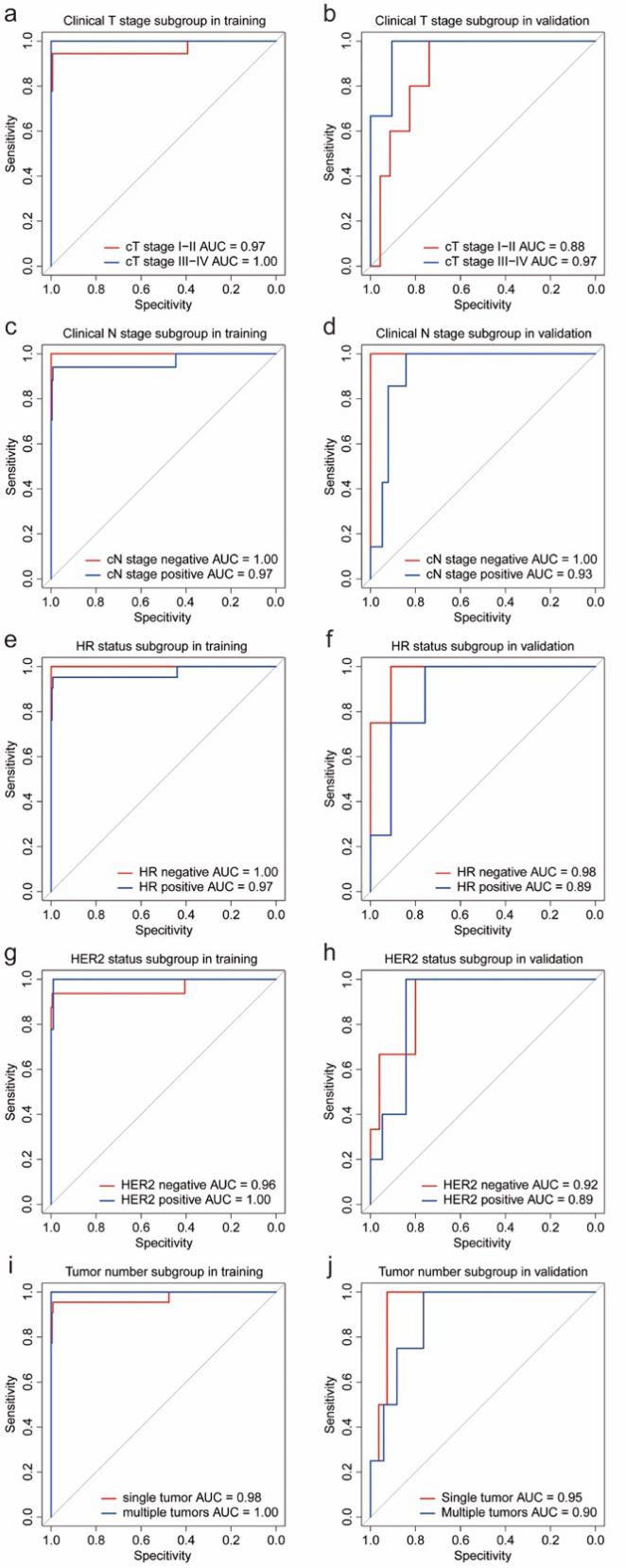


AUC of subgroups stratified by clinical T stage in the training cohort (A) and validation cohort (B). AUC of subgroups stratified by clinical N stage in the training cohort (C) and validation cohort (D). AUC of subgroups stratified by HR status in the training cohort (E) and validation cohort (F). AUC of subgroups stratified by HER-2 status in the training cohort (G) and validation cohort (H). AUC of subgroups stratified by tumor number (n=1 vs n>1) in the training cohort (I) and validation cohort (J). AUC, area under curve; T, tumor; N, node; HR, hormone receptor; Her2, human epidermal growth factor receptor 2.

**Figure S3. Comparison of the performance of clinical model and tumoral + peritumoral multiparametric MRI radiomics model**


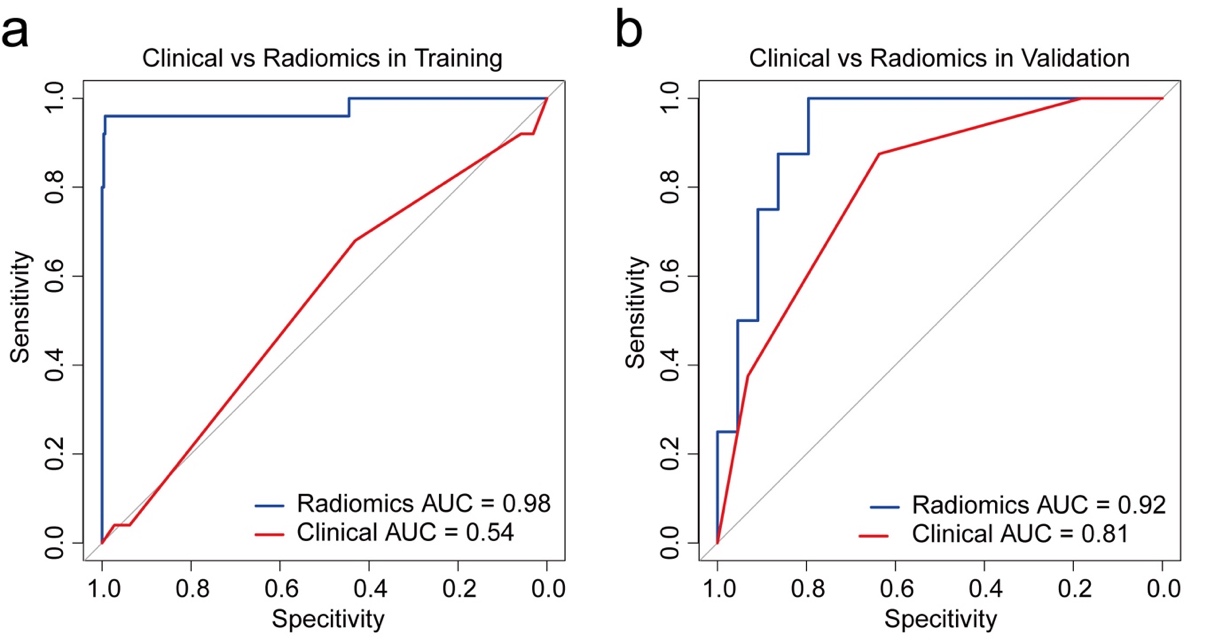


AUC of clinical model and tumoral + peritumoral multiparametric MRI radiomics model in the training cohort (A) and validation cohort (B). AUC, area under curve.

**Table S1. Magnetic resonance imaging scanning parameters for the patients**

| **Hospital** | **Scanner** | **Sequence** | **TR/TE**  **(ms)** | **FOV**  **(mm)** | **Matrix** | **Slice Thickness (mm)** | **Slice Gap**  **(mm)** | **Slices** | **Flip Angle** | **Acquisition**  **Time**  **(min)** | **Scans** |
| --- | --- | --- | --- | --- | --- | --- | --- | --- | --- | --- | --- |
| **SYSMH** | Philips 1·5T  (Achieva) | T2WI | 4000/60 | 337×240 | 400×318 | 3 | 0 | 55 | 90° | 4min |  |
|  |  | DWI-ADC | 7439/53 | 363×340 | 360×301 | 3 | 0 | 55 | 90° | 4min35s |  |
|  |  | T1+C | 3·3/1·54 | 320×250 | 217×172 | 1 | 0 | 55 | 10° | 7min | 55 |
|  | Philips 3·0T  (Ingenia) | T2WI | 4000/60 | 337×240 | 400×318 | 3 | 0 | 55 | 90° | 4min |  |
|  |  | DWI-ADC | 7439/53 | 363×340 | 360×301 | 3 | 0 | 55 | 90° | 4min35s |  |
|  |  | T1+C | 3·3/1·54 | 320×250 | 217×172 | 1 | 0 | 55 | 10° | 7min | 55 |
|  | Siemens 1·5T  (Avanto) | T2WI | 2760/107 | 350×350 | 320×224 | 5 | 1 | 30 | 150° | 2min46s |  |
|  |  | DWI-ADC | 5400/119 | 400×252 | 200×170 | 6 | 1·8 | 20 | 180° | 2min34s |  |
|  |  | T1+C | 4·95/2·2 | 380×269 | 288×216 | 3 | 0·6 | 48-72 | 10° | 5-7min | 50/70 |
|  | Siemens 3·0T  (Skyra) | T2WI | 7600/75 | 340×340 | 448×358 | 4 | 0·8 | 35 | 116° | 3min42s |  |
|  |  | DWI-ADC | 7620/64 | 360×310 | 192×192 | 4 | 0·8 | 35 | 180° | 1min54s |  |
|  |  | T1+C | 3·25/1·22 | 380×327 | 256×218 | 2·5 | 0·5 | 48-72 | 10° | 5-7min | 50/70 |
| **SYSUCC** | United Imaging 3·0T  (China) | T2WI | 3600/74·34 | 340×340 | 336×335 | 5 | 1 | 24 | 90° | 3min05s |  |
|  |  | DWI-ADC | 3597/67·2 | 350×350 | 350×190 | 6 | 1 | 24 | 90° | 1min33s |  |
|  |  | T1+C | 4·3/1·99 | 340×340 | 336×335 | 0·67 | 0 | 204 | 10° | 9min58s | 8 |
|  | GE 3·0T  (USA) | T2WI | 3912/107·64 | 380×380 | 416×256 | 5 | 1 | 28 | 111° | 1min59s |  |
|  |  | DWI-ADC | 4168/60·2 | 380×380 | 128×160 | 5 | 1 | 48 | - | 1min40s |  |
|  |  | T1+C | 4·3/1·7 | 360×360 | 256×320 | 1 | 0 | 204 | 5° | 9min50s | 8 |

Abbreviations: FOV, field of view; TR, repetition time; TE, echo time; T1+C, contrast-enhanced T1-weighted imaging; T2WI, T2-weighted imaging; DWI-ADC, diffusion-weighted imaging quantitatively measured apparent diffusion coefficients; SYSMH, Sun Yat-sen Memorial Hospital of Sun Yat-sen University; SYSUCC, Sun Yat-sen University Cancer center.

**Table S2. The radiomic features extracted via 3D Slicer PyRadiomics**

|  | Image type | Feature Class | Feature Name |
| --- | --- | --- | --- |
|  | diagnostics | Versions | PyRadiomics |
| 1 | diagnostics | Image-original | Mean |
| 2 | diagnostics | Image-original | Maximum |
| 3 | diagnostics | Mask-original | VoxelNum |
| 4 | diagnostics | Mask-original | VolumeNum |
| 5 | diagnostics | Image-interpolated | Mean |
| 6 | diagnostics | Image-interpolated | Minimum |
| 7 | diagnostics | Image-interpolated | Maximum |
| 8 | diagnostics | Mask-interpolated | VoxelNum |
| 9 | diagnostics | Mask-interpolated | VolumeNum |
| 10 | diagnostics | Mask-interpolated | Mean |
| 11 | diagnostics | Mask-interpolated | Minimum |
| 12 | diagnostics | Mask-interpolated | Maximum |
| 13 | original | shape | VoxelVolume |
| 14 | original | shape | Maximum3DDiameter |
| 15 | original | shape | MeshVolume |
| 16 | original | shape | MajorAxisLength |
| 17 | original | shape | Sphericity |
| 18 | original | shape | LeastAxisLength |
| 19 | original | shape | Elongation |
| 20 | original | shape | SurfaceVolumeRatio |
| 21 | original | shape | Maximum2DDiameterSlice |
| 22 | original | shape | Flatness |
| 23 | original | shape | SurfaceArea |
| 24 | original | shape | MinorAxisLength |
| 25 | original | shape | Maximum2DDiameterColumn |
| 26 | original | shape | Maximum2DDiameterRow |
| 27 | original | gldm | GrayLevelVariance |
| 28 | original | gldm | HighGrayLevelEmphasis |
| 29 | original | gldm | DependenceEntropy |
| 30 | original | gldm | DependenceNonUniformity |
| 31 | original | gldm | GrayLevelNonUniformity |
| 32 | original | gldm | SmallDependenceEmphasis |
| 33 | original | gldm | SmallDependenceHighGrayLevelEmphasis |
| 34 | original | gldm | DependenceNonUniformityNormalized |
| 35 | original | gldm | LargeDependenceEmphasis |
| 36 | original | gldm | LargeDependenceLowGrayLevelEmphasis |
| 37 | original | gldm | DependenceVariance |
| 38 | original | gldm | LargeDependenceHighGrayLevelEmphasis |
| 39 | original | gldm | SmallDependenceLowGrayLevelEmphasis |
| 40 | original | gldm | LowGrayLevelEmphasis |
| 41 | original | glcm | JointAverage |
| 42 | original | glcm | SumAverage |
| 43 | original | glcm | JointEntropy |
| 44 | original | glcm | ClusterShade |
| 45 | original | glcm | MaximumProbability |
| 46 | original | glcm | Idmn |
| 47 | original | glcm | JointEnergy |
| 48 | original | glcm | Contrast |
| 49 | original | glcm | DifferenceEntropy |
| 50 | original | glcm | InverseVariance |
| 51 | original | glcm | DifferenceVariance |
| 52 | original | glcm | Idn |
| 53 | original | glcm | Idm |
| 54 | original | glcm | Correlation |
| 55 | original | glcm | Autocorrelation |
| 56 | original | glcm | SumEntropy |
| 57 | original | glcm | MCC |
| 58 | original | glcm | SumSquares |
| 59 | original | glcm | ClusterProminence |
| 60 | original | glcm | Imc2 |
| 61 | original | glcm | Imc1 |
| 62 | original | glcm | DifferenceAverage |
| 63 | original | glcm | Id |
| 64 | original | glcm | ClusterTendency |
| 65 | original | firstorder | InterquartileRange |
| 66 | original | firstorder | Skewness |
| 67 | original | firstorder | Uniformity |
| 68 | original | firstorder | Median |
| 69 | original | firstorder | Energy |
| 70 | original | firstorder | RobustMeanAbsoluteDeviation |
| 71 | original | firstorder | MeanAbsoluteDeviation |
| 72 | original | firstorder | TotalEnergy |
| 73 | original | firstorder | Maximum |
| 74 | original | firstorder | RootMeanSquared |
| 75 | original | firstorder | 90Percentile |
| 76 | original | firstorder | Minimum |
| 77 | original | firstorder | Entropy |
| 78 | original | firstorder | Range |
| 79 | original | firstorder | Variance |
| 80 | original | firstorder | 10Percentile |
| 81 | original | firstorder | Kurtosis |
| 82 | original | firstorder | Mean |
| 83 | original | glrlm | ShortRunLowGrayLevelEmphasis |
| 84 | original | glrlm | GrayLevelVariance |
| 85 | original | glrlm | LowGrayLevelRunEmphasis |
| 86 | original | glrlm | GrayLevelNonUniformityNormalized |
| 87 | original | glrlm | RunVariance |
| 88 | original | glrlm | GrayLevelNonUniformity |
| 89 | original | glrlm | LongRunEmphasis |
| 90 | original | glrlm | ShortRunHighGrayLevelEmphasis |
| 91 | original | glrlm | RunLengthNonUniformity |
| 92 | original | glrlm | ShortRunEmphasis |
| 93 | original | glrlm | LongRunHighGrayLevelEmphasis |
| 94 | original | glrlm | RunPercentage |
| 95 | original | glrlm | LongRunLowGrayLevelEmphasis |
| 96 | original | glrlm | RunEntropy |
| 97 | original | glrlm | HighGrayLevelRunEmphasis |
| 98 | original | glrlm | RunLengthNonUniformityNormalized |
| 99 | original | glszm | GrayLevelVariance |
| 100 | original | glszm | ZoneVariance |
| 101 | original | glszm | GrayLevelNonUniformityNormalized |
| 102 | original | glszm | SizeZoneNonUniformityNormalized |
| 103 | original | glszm | SizeZoneNonUniformity |
| 104 | original | glszm | GrayLevelNonUniformity |
| 105 | original | glszm | LargeAreaEmphasis |
| 106 | original | glszm | SmallAreaHighGrayLevelEmphasis |
| 107 | original | glszm | ZonePercentage |
| 108 | original | glszm | LargeAreaLowGrayLevelEmphasis |
| 109 | original | glszm | LargeAreaHighGrayLevelEmphasis |
| 110 | original | glszm | HighGrayLevelZoneEmphasis |
| 111 | original | glszm | SmallAreaEmphasis |
| 112 | original | glszm | LowGrayLevelZoneEmphasis |
| 113 | original | glszm | ZoneEntropy |
| 114 | original | glszm | SmallAreaLowGrayLevelEmphasis |
| 115 | original | ngtdm | Coarseness |
| 116 | original | ngtdm | Complexity |
| 117 | original | ngtdm | Strength |
| 118 | original | ngtdm | Contrast |
| 119 | original | ngtdm | Busyness |
| 120 | wavelet-HLL | gldm | GrayLevelVariance |
| 121 | wavelet-HLL | gldm | HighGrayLevelEmphasis |
| 122 | wavelet-HLL | gldm | DependenceEntropy |
| 123 | wavelet-HLL | gldm | DependenceNonUniformity |
| 124 | wavelet-HLL | gldm | GrayLevelNonUniformity |
| 125 | wavelet-HLL | gldm | SmallDependenceEmphasis |
| 126 | wavelet-HLL | gldm | SmallDependenceHighGrayLevelEmphasis |
| 127 | wavelet-HLL | gldm | DependenceNonUniformityNormalized |
| 128 | wavelet-HLL | gldm | LargeDependenceEmphasis |
| 129 | wavelet-HLL | gldm | LargeDependenceLowGrayLevelEmphasis |
| 130 | wavelet-HLL | gldm | DependenceVariance |
| 131 | wavelet-HLL | gldm | LargeDependenceHighGrayLevelEmphasis |
| 132 | wavelet-HLL | gldm | SmallDependenceLowGrayLevelEmphasis |
| 133 | wavelet-HLL | gldm | LowGrayLevelEmphasis |
| 134 | wavelet-HLL | glcm | JointAverage |
| 135 | wavelet-HLL | glcm | SumAverage |
| 136 | wavelet-HLL | glcm | JointEntropy |
| 137 | wavelet-HLL | glcm | ClusterShade |
| 138 | wavelet-HLL | glcm | MaximumProbability |
| 139 | wavelet-HLL | glcm | Idmn |
| 140 | wavelet-HLL | glcm | JointEnergy |
| 141 | wavelet-HLL | glcm | Contrast |
| 142 | wavelet-HLL | glcm | DifferenceEntropy |
| 143 | wavelet-HLL | glcm | InverseVariance |
| 144 | wavelet-HLL | glcm | DifferenceVariance |
| 145 | wavelet-HLL | glcm | Idn |
| 146 | wavelet-HLL | glcm | Idm |
| 147 | wavelet-HLL | glcm | Correlation |
| 148 | wavelet-HLL | glcm | Autocorrelation |
| 149 | wavelet-HLL | glcm | SumEntropy |
| 150 | wavelet-HLL | glcm | MCC |
| 151 | wavelet-HLL | glcm | SumSquares |
| 152 | wavelet-HLL | glcm | ClusterProminence |
| 153 | wavelet-HLL | glcm | Imc2 |
| 154 | wavelet-HLL | glcm | Imc1 |
| 155 | wavelet-HLL | glcm | DifferenceAverage |
| 156 | wavelet-HLL | glcm | Id |
| 157 | wavelet-HLL | glcm | ClusterTendency |
| 158 | wavelet-HLL | firstorder | InterquartileRange |
| 159 | wavelet-HLL | firstorder | Skewness |
| 160 | wavelet-HLL | firstorder | Uniformity |
| 161 | wavelet-HLL | firstorder | Median |
| 162 | wavelet-HLL | firstorder | Energy |
| 163 | wavelet-HLL | firstorder | RobustMeanAbsoluteDeviation |
| 164 | wavelet-HLL | firstorder | MeanAbsoluteDeviation |
| 165 | wavelet-HLL | firstorder | TotalEnergy |
| 166 | wavelet-HLL | firstorder | Maximum |
| 167 | wavelet-HLL | firstorder | RootMeanSquared |
| 168 | wavelet-HLL | firstorder | 90Percentile |
| 169 | wavelet-HLL | firstorder | Minimum |
| 170 | wavelet-HLL | firstorder | Entropy |
| 171 | wavelet-HLL | firstorder | Range |
| 172 | wavelet-HLL | firstorder | Variance |
| 173 | wavelet-HLL | firstorder | 10Percentile |
| 174 | wavelet-HLL | firstorder | Kurtosis |
| 175 | wavelet-HLL | firstorder | Mean |
| 176 | wavelet-HLL | glrlm | ShortRunLowGrayLevelEmphasis |
| 177 | wavelet-HLL | glrlm | GrayLevelVariance |
| 178 | wavelet-HLL | glrlm | LowGrayLevelRunEmphasis |
| 179 | wavelet-HLL | glrlm | GrayLevelNonUniformityNormalized |
| 180 | wavelet-HLL | glrlm | RunVariance |
| 181 | wavelet-HLL | glrlm | GrayLevelNonUniformity |
| 182 | wavelet-HLL | glrlm | LongRunEmphasis |
| 183 | wavelet-HLL | glrlm | ShortRunHighGrayLevelEmphasis |
| 184 | wavelet-HLL | glrlm | RunLengthNonUniformity |
| 185 | wavelet-HLL | glrlm | ShortRunEmphasis |
| 186 | wavelet-HLL | glrlm | LongRunHighGrayLevelEmphasis |
| 187 | wavelet-HLL | glrlm | RunPercentage |
| 188 | wavelet-HLL | glrlm | LongRunLowGrayLevelEmphasis |
| 189 | wavelet-HLL | glrlm | RunEntropy |
| 190 | wavelet-HLL | glrlm | HighGrayLevelRunEmphasis |
| 191 | wavelet-HLL | glrlm | RunLengthNonUniformityNormalized |
| 192 | wavelet-HLL | glszm | GrayLevelVariance |
| 193 | wavelet-HLL | glszm | ZoneVariance |
| 194 | wavelet-HLL | glszm | GrayLevelNonUniformityNormalized |
| 195 | wavelet-HLL | glszm | SizeZoneNonUniformityNormalized |
| 196 | wavelet-HLL | glszm | SizeZoneNonUniformity |
| 197 | wavelet-HLL | glszm | GrayLevelNonUniformity |
| 198 | wavelet-HLL | glszm | LargeAreaEmphasis |
| 199 | wavelet-HLL | glszm | SmallAreaHighGrayLevelEmphasis |
| 200 | wavelet-HLL | glszm | ZonePercentage |
| 201 | wavelet-HLL | glszm | LargeAreaLowGrayLevelEmphasis |
| 202 | wavelet-HLL | glszm | LargeAreaHighGrayLevelEmphasis |
| 203 | wavelet-HLL | glszm | HighGrayLevelZoneEmphasis |
| 204 | wavelet-HLL | glszm | SmallAreaEmphasis |
| 205 | wavelet-HLL | glszm | LowGrayLevelZoneEmphasis |
| 206 | wavelet-HLL | glszm | ZoneEntropy |
| 207 | wavelet-HLL | glszm | SmallAreaLowGrayLevelEmphasis |
| 208 | wavelet-HLL | ngtdm | Coarseness |
| 209 | wavelet-HLL | ngtdm | Complexity |
| 210 | wavelet-HLL | ngtdm | Strength |
| 211 | wavelet-HLL | ngtdm | Contrast |
| 212 | wavelet-HLL | ngtdm | Busyness |
| 213 | wavelet-LHL | gldm | GrayLevelVariance |
| 214 | wavelet-LHL | gldm | HighGrayLevelEmphasis |
| 215 | wavelet-LHL | gldm | DependenceEntropy |
| 216 | wavelet-LHL | gldm | DependenceNonUniformity |
| 217 | wavelet-LHL | gldm | GrayLevelNonUniformity |
| 218 | wavelet-LHL | gldm | SmallDependenceEmphasis |
| 219 | wavelet-LHL | gldm | SmallDependenceHighGrayLevelEmphasis |
| 220 | wavelet-LHL | gldm | DependenceNonUniformityNormalized |
| 221 | wavelet-LHL | gldm | LargeDependenceEmphasis |
| 222 | wavelet-LHL | gldm | LargeDependenceLowGrayLevelEmphasis |
| 223 | wavelet-LHL | gldm | DependenceVariance |
| 224 | wavelet-LHL | gldm | LargeDependenceHighGrayLevelEmphasis |
| 225 | wavelet-LHL | gldm | SmallDependenceLowGrayLevelEmphasis |
| 226 | wavelet-LHL | gldm | LowGrayLevelEmphasis |
| 227 | wavelet-LHL | glcm | JointAverage |
| 228 | wavelet-LHL | glcm | SumAverage |
| 229 | wavelet-LHL | glcm | JointEntropy |
| 230 | wavelet-LHL | glcm | ClusterShade |
| 231 | wavelet-LHL | glcm | MaximumProbability |
| 232 | wavelet-LHL | glcm | Idmn |
| 233 | wavelet-LHL | glcm | JointEnergy |
| 234 | wavelet-LHL | glcm | Contrast |
| 235 | wavelet-LHL | glcm | DifferenceEntropy |
| 236 | wavelet-LHL | glcm | InverseVariance |
| 237 | wavelet-LHL | glcm | DifferenceVariance |
| 238 | wavelet-LHL | glcm | Idn |
| 239 | wavelet-LHL | glcm | Idm |
| 240 | wavelet-LHL | glcm | Correlation |
| 241 | wavelet-LHL | glcm | Autocorrelation |
| 242 | wavelet-LHL | glcm | SumEntropy |
| 243 | wavelet-LHL | glcm | MCC |
| 244 | wavelet-LHL | glcm | SumSquares |
| 245 | wavelet-LHL | glcm | ClusterProminence |
| 246 | wavelet-LHL | glcm | Imc2 |
| 247 | wavelet-LHL | glcm | Imc1 |
| 248 | wavelet-LHL | glcm | DifferenceAverage |
| 249 | wavelet-LHL | glcm | Id |
| 250 | wavelet-LHL | glcm | ClusterTendency |
| 251 | wavelet-LHL | firstorder | InterquartileRange |
| 252 | wavelet-LHL | firstorder | Skewness |
| 253 | wavelet-LHL | firstorder | Uniformity |
| 254 | wavelet-LHL | firstorder | Median |
| 255 | wavelet-LHL | firstorder | Energy |
| 256 | wavelet-LHL | firstorder | RobustMeanAbsoluteDeviation |
| 257 | wavelet-LHL | firstorder | MeanAbsoluteDeviation |
| 258 | wavelet-LHL | firstorder | TotalEnergy |
| 259 | wavelet-LHL | firstorder | Maximum |
| 260 | wavelet-LHL | firstorder | RootMeanSquared |
| 261 | wavelet-LHL | firstorder | 90Percentile |
| 262 | wavelet-LHL | firstorder | Minimum |
| 263 | wavelet-LHL | firstorder | Entropy |
| 264 | wavelet-LHL | firstorder | Range |
| 265 | wavelet-LHL | firstorder | Variance |
| 266 | wavelet-LHL | firstorder | 10Percentile |
| 267 | wavelet-LHL | firstorder | Kurtosis |
| 268 | wavelet-LHL | firstorder | Mean |
| 269 | wavelet-LHL | glrlm | ShortRunLowGrayLevelEmphasis |
| 270 | wavelet-LHL | glrlm | GrayLevelVariance |
| 271 | wavelet-LHL | glrlm | LowGrayLevelRunEmphasis |
| 272 | wavelet-LHL | glrlm | GrayLevelNonUniformityNormalized |
| 273 | wavelet-LHL | glrlm | RunVariance |
| 274 | wavelet-LHL | glrlm | GrayLevelNonUniformity |
| 275 | wavelet-LHL | glrlm | LongRunEmphasis |
| 276 | wavelet-LHL | glrlm | ShortRunHighGrayLevelEmphasis |
| 277 | wavelet-LHL | glrlm | RunLengthNonUniformity |
| 278 | wavelet-LHL | glrlm | ShortRunEmphasis |
| 279 | wavelet-LHL | glrlm | LongRunHighGrayLevelEmphasis |
| 280 | wavelet-LHL | glrlm | RunPercentage |
| 281 | wavelet-LHL | glrlm | LongRunLowGrayLevelEmphasis |
| 282 | wavelet-LHL | glrlm | RunEntropy |
| 283 | wavelet-LHL | glrlm | HighGrayLevelRunEmphasis |
| 284 | wavelet-LHL | glrlm | RunLengthNonUniformityNormalized |
| 285 | wavelet-LHL | glszm | GrayLevelVariance |
| 286 | wavelet-LHL | glszm | ZoneVariance |
| 287 | wavelet-LHL | glszm | GrayLevelNonUniformityNormalized |
| 288 | wavelet-LHL | glszm | SizeZoneNonUniformityNormalized |
| 289 | wavelet-LHL | glszm | SizeZoneNonUniformity |
| 290 | wavelet-LHL | glszm | GrayLevelNonUniformity |
| 291 | wavelet-LHL | glszm | LargeAreaEmphasis |
| 292 | wavelet-LHL | glszm | SmallAreaHighGrayLevelEmphasis |
| 293 | wavelet-LHL | glszm | ZonePercentage |
| 294 | wavelet-LHL | glszm | LargeAreaLowGrayLevelEmphasis |
| 295 | wavelet-LHL | glszm | LargeAreaHighGrayLevelEmphasis |
| 296 | wavelet-LHL | glszm | HighGrayLevelZoneEmphasis |
| 297 | wavelet-LHL | glszm | SmallAreaEmphasis |
| 298 | wavelet-LHL | glszm | LowGrayLevelZoneEmphasis |
| 299 | wavelet-LHL | glszm | ZoneEntropy |
| 300 | wavelet-LHL | glszm | SmallAreaLowGrayLevelEmphasis |
| 301 | wavelet-LHL | ngtdm | Coarseness |
| 302 | wavelet-LHL | ngtdm | Complexity |
| 303 | wavelet-LHL | ngtdm | Strength |
| 304 | wavelet-LHL | ngtdm | Contrast |
| 305 | wavelet-LHL | ngtdm | Busyness |
| 306 | wavelet-LHH | gldm | GrayLevelVariance |
| 307 | wavelet-LHH | gldm | HighGrayLevelEmphasis |
| 308 | wavelet-LHH | gldm | DependenceEntropy |
| 309 | wavelet-LHH | gldm | DependenceNonUniformity |
| 310 | wavelet-LHH | gldm | GrayLevelNonUniformity |
| 311 | wavelet-LHH | gldm | SmallDependenceEmphasis |
| 312 | wavelet-LHH | gldm | SmallDependenceHighGrayLevelEmphasis |
| 313 | wavelet-LHH | gldm | DependenceNonUniformityNormalized |
| 314 | wavelet-LHH | gldm | LargeDependenceEmphasis |
| 315 | wavelet-LHH | gldm | LargeDependenceLowGrayLevelEmphasis |
| 316 | wavelet-LHH | gldm | DependenceVariance |
| 317 | wavelet-LHH | gldm | LargeDependenceHighGrayLevelEmphasis |
| 318 | wavelet-LHH | gldm | SmallDependenceLowGrayLevelEmphasis |
| 319 | wavelet-LHH | gldm | LowGrayLevelEmphasis |
| 320 | wavelet-LHH | glcm | JointAverage |
| 321 | wavelet-LHH | glcm | SumAverage |
| 322 | wavelet-LHH | glcm | JointEntropy |
| 323 | wavelet-LHH | glcm | ClusterShade |
| 324 | wavelet-LHH | glcm | MaximumProbability |
| 325 | wavelet-LHH | glcm | Idmn |
| 326 | wavelet-LHH | glcm | JointEnergy |
| 327 | wavelet-LHH | glcm | Contrast |
| 328 | wavelet-LHH | glcm | DifferenceEntropy |
| 329 | wavelet-LHH | glcm | InverseVariance |
| 330 | wavelet-LHH | glcm | DifferenceVariance |
| 331 | wavelet-LHH | glcm | Idn |
| 332 | wavelet-LHH | glcm | Idm |
| 333 | wavelet-LHH | glcm | Correlation |
| 334 | wavelet-LHH | glcm | Autocorrelation |
| 335 | wavelet-LHH | glcm | SumEntropy |
| 336 | wavelet-LHH | glcm | MCC |
| 337 | wavelet-LHH | glcm | SumSquares |
| 338 | wavelet-LHH | glcm | ClusterProminence |
| 339 | wavelet-LHH | glcm | Imc2 |
| 340 | wavelet-LHH | glcm | Imc1 |
| 341 | wavelet-LHH | glcm | DifferenceAverage |
| 342 | wavelet-LHH | glcm | Id |
| 343 | wavelet-LHH | glcm | ClusterTendency |
| 344 | wavelet-LHH | firstorder | InterquartileRange |
| 345 | wavelet-LHH | firstorder | Skewness |
| 346 | wavelet-LHH | firstorder | Uniformity |
| 347 | wavelet-LHH | firstorder | Median |
| 348 | wavelet-LHH | firstorder | Energy |
| 349 | wavelet-LHH | firstorder | RobustMeanAbsoluteDeviation |
| 350 | wavelet-LHH | firstorder | MeanAbsoluteDeviation |
| 351 | wavelet-LHH | firstorder | TotalEnergy |
| 352 | wavelet-LHH | firstorder | Maximum |
| 353 | wavelet-LHH | firstorder | RootMeanSquared |
| 354 | wavelet-LHH | firstorder | 90Percentile |
| 355 | wavelet-LHH | firstorder | Minimum |
| 356 | wavelet-LHH | firstorder | Entropy |
| 357 | wavelet-LHH | firstorder | Range |
| 358 | wavelet-LHH | firstorder | Variance |
| 359 | wavelet-LHH | firstorder | 10Percentile |
| 360 | wavelet-LHH | firstorder | Kurtosis |
| 361 | wavelet-LHH | firstorder | Mean |
| 362 | wavelet-LHH | glrlm | ShortRunLowGrayLevelEmphasis |
| 363 | wavelet-LHH | glrlm | GrayLevelVariance |
| 364 | wavelet-LHH | glrlm | LowGrayLevelRunEmphasis |
| 365 | wavelet-LHH | glrlm | GrayLevelNonUniformityNormalized |
| 366 | wavelet-LHH | glrlm | RunVariance |
| 367 | wavelet-LHH | glrlm | GrayLevelNonUniformity |
| 368 | wavelet-LHH | glrlm | LongRunEmphasis |
| 369 | wavelet-LHH | glrlm | ShortRunHighGrayLevelEmphasis |
| 370 | wavelet-LHH | glrlm | RunLengthNonUniformity |
| 371 | wavelet-LHH | glrlm | ShortRunEmphasis |
| 372 | wavelet-LHH | glrlm | LongRunHighGrayLevelEmphasis |
| 373 | wavelet-LHH | glrlm | RunPercentage |
| 374 | wavelet-LHH | glrlm | LongRunLowGrayLevelEmphasis |
| 375 | wavelet-LHH | glrlm | RunEntropy |
| 376 | wavelet-LHH | glrlm | HighGrayLevelRunEmphasis |
| 377 | wavelet-LHH | glrlm | RunLengthNonUniformityNormalized |
| 378 | wavelet-LHH | glszm | GrayLevelVariance |
| 379 | wavelet-LHH | glszm | ZoneVariance |
| 380 | wavelet-LHH | glszm | GrayLevelNonUniformityNormalized |
| 381 | wavelet-LHH | glszm | SizeZoneNonUniformityNormalized |
| 382 | wavelet-LHH | glszm | SizeZoneNonUniformity |
| 383 | wavelet-LHH | glszm | GrayLevelNonUniformity |
| 384 | wavelet-LHH | glszm | LargeAreaEmphasis |
| 385 | wavelet-LHH | glszm | SmallAreaHighGrayLevelEmphasis |
| 386 | wavelet-LHH | glszm | ZonePercentage |
| 387 | wavelet-LHH | glszm | LargeAreaLowGrayLevelEmphasis |
| 388 | wavelet-LHH | glszm | LargeAreaHighGrayLevelEmphasis |
| 389 | wavelet-LHH | glszm | HighGrayLevelZoneEmphasis |
| 390 | wavelet-LHH | glszm | SmallAreaEmphasis |
| 391 | wavelet-LHH | glszm | LowGrayLevelZoneEmphasis |
| 392 | wavelet-LHH | glszm | ZoneEntropy |
| 393 | wavelet-LHH | glszm | SmallAreaLowGrayLevelEmphasis |
| 394 | wavelet-LHH | ngtdm | Coarseness |
| 395 | wavelet-LHH | ngtdm | Complexity |
| 396 | wavelet-LHH | ngtdm | Strength |
| 397 | wavelet-LHH | ngtdm | Contrast |
| 398 | wavelet-LHH | ngtdm | Busyness |
| 399 | wavelet-LLH | gldm | GrayLevelVariance |
| 400 | wavelet-LLH | gldm | HighGrayLevelEmphasis |
| 401 | wavelet-LLH | gldm | DependenceEntropy |
| 402 | wavelet-LLH | gldm | DependenceNonUniformity |
| 403 | wavelet-LLH | gldm | GrayLevelNonUniformity |
| 404 | wavelet-LLH | gldm | SmallDependenceEmphasis |
| 405 | wavelet-LLH | gldm | SmallDependenceHighGrayLevelEmphasis |
| 406 | wavelet-LLH | gldm | DependenceNonUniformityNormalized |
| 407 | wavelet-LLH | gldm | LargeDependenceEmphasis |
| 408 | wavelet-LLH | gldm | LargeDependenceLowGrayLevelEmphasis |
| 409 | wavelet-LLH | gldm | DependenceVariance |
| 410 | wavelet-LLH | gldm | LargeDependenceHighGrayLevelEmphasis |
| 411 | wavelet-LLH | gldm | SmallDependenceLowGrayLevelEmphasis |
| 412 | wavelet-LLH | gldm | LowGrayLevelEmphasis |
| 413 | wavelet-LLH | glcm | JointAverage |
| 414 | wavelet-LLH | glcm | SumAverage |
| 415 | wavelet-LLH | glcm | JointEntropy |
| 416 | wavelet-LLH | glcm | ClusterShade |
| 417 | wavelet-LLH | glcm | MaximumProbability |
| 418 | wavelet-LLH | glcm | Idmn |
| 419 | wavelet-LLH | glcm | JointEnergy |
| 420 | wavelet-LLH | glcm | Contrast |
| 421 | wavelet-LLH | glcm | DifferenceEntropy |
| 422 | wavelet-LLH | glcm | InverseVariance |
| 423 | wavelet-LLH | glcm | DifferenceVariance |
| 424 | wavelet-LLH | glcm | Idn |
| 425 | wavelet-LLH | glcm | Idm |
| 426 | wavelet-LLH | glcm | Correlation |
| 427 | wavelet-LLH | glcm | Autocorrelation |
| 428 | wavelet-LLH | glcm | SumEntropy |
| 429 | wavelet-LLH | glcm | MCC |
| 430 | wavelet-LLH | glcm | SumSquares |
| 431 | wavelet-LLH | glcm | ClusterProminence |
| 432 | wavelet-LLH | glcm | Imc2 |
| 433 | wavelet-LLH | glcm | Imc1 |
| 434 | wavelet-LLH | glcm | DifferenceAverage |
| 435 | wavelet-LLH | glcm | Id |
| 436 | wavelet-LLH | glcm | ClusterTendency |
| 437 | wavelet-LLH | firstorder | InterquartileRange |
| 438 | wavelet-LLH | firstorder | Skewness |
| 439 | wavelet-LLH | firstorder | Uniformity |
| 440 | wavelet-LLH | firstorder | Median |
| 441 | wavelet-LLH | firstorder | Energy |
| 442 | wavelet-LLH | firstorder | RobustMeanAbsoluteDeviation |
| 443 | wavelet-LLH | firstorder | MeanAbsoluteDeviation |
| 444 | wavelet-LLH | firstorder | TotalEnergy |
| 445 | wavelet-LLH | firstorder | Maximum |
| 446 | wavelet-LLH | firstorder | RootMeanSquared |
| 447 | wavelet-LLH | firstorder | 90Percentile |
| 448 | wavelet-LLH | firstorder | Minimum |
| 449 | wavelet-LLH | firstorder | Entropy |
| 450 | wavelet-LLH | firstorder | Range |
| 451 | wavelet-LLH | firstorder | Variance |
| 452 | wavelet-LLH | firstorder | 10Percentile |
| 453 | wavelet-LLH | firstorder | Kurtosis |
| 454 | wavelet-LLH | firstorder | Mean |
| 455 | wavelet-LLH | glrlm | ShortRunLowGrayLevelEmphasis |
| 456 | wavelet-LLH | glrlm | GrayLevelVariance |
| 457 | wavelet-LLH | glrlm | LowGrayLevelRunEmphasis |
| 458 | wavelet-LLH | glrlm | GrayLevelNonUniformityNormalized |
| 459 | wavelet-LLH | glrlm | RunVariance |
| 460 | wavelet-LLH | glrlm | GrayLevelNonUniformity |
| 461 | wavelet-LLH | glrlm | LongRunEmphasis |
| 462 | wavelet-LLH | glrlm | ShortRunHighGrayLevelEmphasis |
| 463 | wavelet-LLH | glrlm | RunLengthNonUniformity |
| 464 | wavelet-LLH | glrlm | ShortRunEmphasis |
| 465 | wavelet-LLH | glrlm | LongRunHighGrayLevelEmphasis |
| 466 | wavelet-LLH | glrlm | RunPercentage |
| 467 | wavelet-LLH | glrlm | LongRunLowGrayLevelEmphasis |
| 468 | wavelet-LLH | glrlm | RunEntropy |
| 469 | wavelet-LLH | glrlm | HighGrayLevelRunEmphasis |
| 470 | wavelet-LLH | glrlm | RunLengthNonUniformityNormalized |
| 471 | wavelet-LLH | glszm | GrayLevelVariance |
| 472 | wavelet-LLH | glszm | ZoneVariance |
| 473 | wavelet-LLH | glszm | GrayLevelNonUniformityNormalized |
| 474 | wavelet-LLH | glszm | SizeZoneNonUniformityNormalized |
| 475 | wavelet-LLH | glszm | SizeZoneNonUniformity |
| 476 | wavelet-LLH | glszm | GrayLevelNonUniformity |
| 477 | wavelet-LLH | glszm | LargeAreaEmphasis |
| 478 | wavelet-LLH | glszm | SmallAreaHighGrayLevelEmphasis |
| 479 | wavelet-LLH | glszm | ZonePercentage |
| 480 | wavelet-LLH | glszm | LargeAreaLowGrayLevelEmphasis |
| 481 | wavelet-LLH | glszm | LargeAreaHighGrayLevelEmphasis |
| 482 | wavelet-LLH | glszm | HighGrayLevelZoneEmphasis |
| 483 | wavelet-LLH | glszm | SmallAreaEmphasis |
| 484 | wavelet-LLH | glszm | LowGrayLevelZoneEmphasis |
| 485 | wavelet-LLH | glszm | ZoneEntropy |
| 486 | wavelet-LLH | glszm | SmallAreaLowGrayLevelEmphasis |
| 487 | wavelet-LLH | ngtdm | Coarseness |
| 488 | wavelet-LLH | ngtdm | Complexity |
| 489 | wavelet-LLH | ngtdm | Strength |
| 490 | wavelet-LLH | ngtdm | Contrast |
| 491 | wavelet-LLH | ngtdm | Busyness |
| 492 | wavelet-HLH | gldm | GrayLevelVariance |
| 493 | wavelet-HLH | gldm | HighGrayLevelEmphasis |
| 494 | wavelet-HLH | gldm | DependenceEntropy |
| 495 | wavelet-HLH | gldm | DependenceNonUniformity |
| 496 | wavelet-HLH | gldm | GrayLevelNonUniformity |
| 497 | wavelet-HLH | gldm | SmallDependenceEmphasis |
| 498 | wavelet-HLH | gldm | SmallDependenceHighGrayLevelEmphasis |
| 499 | wavelet-HLH | gldm | DependenceNonUniformityNormalized |
| 500 | wavelet-HLH | gldm | LargeDependenceEmphasis |
| 501 | wavelet-HLH | gldm | LargeDependenceLowGrayLevelEmphasis |
| 502 | wavelet-HLH | gldm | DependenceVariance |
| 503 | wavelet-HLH | gldm | LargeDependenceHighGrayLevelEmphasis |
| 504 | wavelet-HLH | gldm | SmallDependenceLowGrayLevelEmphasis |
| 505 | wavelet-HLH | gldm | LowGrayLevelEmphasis |
| 506 | wavelet-HLH | glcm | JointAverage |
| 507 | wavelet-HLH | glcm | SumAverage |
| 508 | wavelet-HLH | glcm | JointEntropy |
| 509 | wavelet-HLH | glcm | ClusterShade |
| 510 | wavelet-HLH | glcm | MaximumProbability |
| 511 | wavelet-HLH | glcm | Idmn |
| 512 | wavelet-HLH | glcm | JointEnergy |
| 513 | wavelet-HLH | glcm | Contrast |
| 514 | wavelet-HLH | glcm | DifferenceEntropy |
| 515 | wavelet-HLH | glcm | InverseVariance |
| 516 | wavelet-HLH | glcm | DifferenceVariance |
| 517 | wavelet-HLH | glcm | Idn |
| 518 | wavelet-HLH | glcm | Idm |
| 519 | wavelet-HLH | glcm | Correlation |
| 520 | wavelet-HLH | glcm | Autocorrelation |
| 521 | wavelet-HLH | glcm | SumEntropy |
| 522 | wavelet-HLH | glcm | MCC |
| 523 | wavelet-HLH | glcm | SumSquares |
| 524 | wavelet-HLH | glcm | ClusterProminence |
| 525 | wavelet-HLH | glcm | Imc2 |
| 526 | wavelet-HLH | glcm | Imc1 |
| 527 | wavelet-HLH | glcm | DifferenceAverage |
| 528 | wavelet-HLH | glcm | Id |
| 529 | wavelet-HLH | glcm | ClusterTendency |
| 530 | wavelet-HLH | firstorder | InterquartileRange |
| 531 | wavelet-HLH | firstorder | Skewness |
| 532 | wavelet-HLH | firstorder | Uniformity |
| 533 | wavelet-HLH | firstorder | Median |
| 534 | wavelet-HLH | firstorder | Energy |
| 535 | wavelet-HLH | firstorder | RobustMeanAbsoluteDeviation |
| 536 | wavelet-HLH | firstorder | MeanAbsoluteDeviation |
| 537 | wavelet-HLH | firstorder | TotalEnergy |
| 538 | wavelet-HLH | firstorder | Maximum |
| 539 | wavelet-HLH | firstorder | RootMeanSquared |
| 540 | wavelet-HLH | firstorder | 90Percentile |
| 541 | wavelet-HLH | firstorder | Minimum |
| 542 | wavelet-HLH | firstorder | Entropy |
| 543 | wavelet-HLH | firstorder | Range |
| 544 | wavelet-HLH | firstorder | Variance |
| 545 | wavelet-HLH | firstorder | 10Percentile |
| 546 | wavelet-HLH | firstorder | Kurtosis |
| 547 | wavelet-HLH | firstorder | Mean |
| 548 | wavelet-HLH | glrlm | ShortRunLowGrayLevelEmphasis |
| 549 | wavelet-HLH | glrlm | GrayLevelVariance |
| 550 | wavelet-HLH | glrlm | LowGrayLevelRunEmphasis |
| 551 | wavelet-HLH | glrlm | GrayLevelNonUniformityNormalized |
| 552 | wavelet-HLH | glrlm | RunVariance |
| 553 | wavelet-HLH | glrlm | GrayLevelNonUniformity |
| 554 | wavelet-HLH | glrlm | LongRunEmphasis |
| 555 | wavelet-HLH | glrlm | ShortRunHighGrayLevelEmphasis |
| 556 | wavelet-HLH | glrlm | RunLengthNonUniformity |
| 557 | wavelet-HLH | glrlm | ShortRunEmphasis |
| 558 | wavelet-HLH | glrlm | LongRunHighGrayLevelEmphasis |
| 559 | wavelet-HLH | glrlm | RunPercentage |
| 560 | wavelet-HLH | glrlm | LongRunLowGrayLevelEmphasis |
| 561 | wavelet-HLH | glrlm | RunEntropy |
| 562 | wavelet-HLH | glrlm | HighGrayLevelRunEmphasis |
| 563 | wavelet-HLH | glrlm | RunLengthNonUniformityNormalized |
| 564 | wavelet-HLH | glszm | GrayLevelVariance |
| 565 | wavelet-HLH | glszm | ZoneVariance |
| 566 | wavelet-HLH | glszm | GrayLevelNonUniformityNormalized |
| 567 | wavelet-HLH | glszm | SizeZoneNonUniformityNormalized |
| 568 | wavelet-HLH | glszm | SizeZoneNonUniformity |
| 569 | wavelet-HLH | glszm | GrayLevelNonUniformity |
| 570 | wavelet-HLH | glszm | LargeAreaEmphasis |
| 571 | wavelet-HLH | glszm | SmallAreaHighGrayLevelEmphasis |
| 572 | wavelet-HLH | glszm | ZonePercentage |
| 573 | wavelet-HLH | glszm | LargeAreaLowGrayLevelEmphasis |
| 574 | wavelet-HLH | glszm | LargeAreaHighGrayLevelEmphasis |
| 575 | wavelet-HLH | glszm | HighGrayLevelZoneEmphasis |
| 576 | wavelet-HLH | glszm | SmallAreaEmphasis |
| 577 | wavelet-HLH | glszm | LowGrayLevelZoneEmphasis |
| 578 | wavelet-HLH | glszm | ZoneEntropy |
| 579 | wavelet-HLH | glszm | SmallAreaLowGrayLevelEmphasis |
| 580 | wavelet-HLH | ngtdm | Coarseness |
| 581 | wavelet-HLH | ngtdm | Complexity |
| 582 | wavelet-HLH | ngtdm | Strength |
| 583 | wavelet-HLH | ngtdm | Contrast |
| 584 | wavelet-HLH | ngtdm | Busyness |
| 585 | wavelet-HHH | gldm | GrayLevelVariance |
| 586 | wavelet-HHH | gldm | HighGrayLevelEmphasis |
| 587 | wavelet-HHH | gldm | DependenceEntropy |
| 588 | wavelet-HHH | gldm | DependenceNonUniformity |
| 589 | wavelet-HHH | gldm | GrayLevelNonUniformity |
| 590 | wavelet-HHH | gldm | SmallDependenceEmphasis |
| 591 | wavelet-HHH | gldm | SmallDependenceHighGrayLevelEmphasis |
| 592 | wavelet-HHH | gldm | DependenceNonUniformityNormalized |
| 593 | wavelet-HHH | gldm | LargeDependenceEmphasis |
| 594 | wavelet-HHH | gldm | LargeDependenceLowGrayLevelEmphasis |
| 595 | wavelet-HHH | gldm | DependenceVariance |
| 596 | wavelet-HHH | gldm | LargeDependenceHighGrayLevelEmphasis |
| 597 | wavelet-HHH | gldm | SmallDependenceLowGrayLevelEmphasis |
| 598 | wavelet-HHH | gldm | LowGrayLevelEmphasis |
| 599 | wavelet-HHH | glcm | JointAverage |
| 600 | wavelet-HHH | glcm | SumAverage |
| 601 | wavelet-HHH | glcm | JointEntropy |
| 602 | wavelet-HHH | glcm | ClusterShade |
| 603 | wavelet-HHH | glcm | MaximumProbability |
| 604 | wavelet-HHH | glcm | Idmn |
| 605 | wavelet-HHH | glcm | JointEnergy |
| 606 | wavelet-HHH | glcm | Contrast |
| 607 | wavelet-HHH | glcm | DifferenceEntropy |
| 608 | wavelet-HHH | glcm | InverseVariance |
| 609 | wavelet-HHH | glcm | DifferenceVariance |
| 610 | wavelet-HHH | glcm | Idn |
| 611 | wavelet-HHH | glcm | Idm |
| 612 | wavelet-HHH | glcm | Correlation |
| 613 | wavelet-HHH | glcm | Autocorrelation |
| 614 | wavelet-HHH | glcm | SumEntropy |
| 615 | wavelet-HHH | glcm | MCC |
| 616 | wavelet-HHH | glcm | SumSquares |
| 617 | wavelet-HHH | glcm | ClusterProminence |
| 618 | wavelet-HHH | glcm | Imc2 |
| 619 | wavelet-HHH | glcm | Imc1 |
| 620 | wavelet-HHH | glcm | DifferenceAverage |
| 621 | wavelet-HHH | glcm | Id |
| 622 | wavelet-HHH | glcm | ClusterTendency |
| 623 | wavelet-HHH | firstorder | InterquartileRange |
| 624 | wavelet-HHH | firstorder | Skewness |
| 625 | wavelet-HHH | firstorder | Uniformity |
| 626 | wavelet-HHH | firstorder | Median |
| 627 | wavelet-HHH | firstorder | Energy |
| 628 | wavelet-HHH | firstorder | RobustMeanAbsoluteDeviation |
| 629 | wavelet-HHH | firstorder | MeanAbsoluteDeviation |
| 630 | wavelet-HHH | firstorder | TotalEnergy |
| 631 | wavelet-HHH | firstorder | Maximum |
| 632 | wavelet-HHH | firstorder | RootMeanSquared |
| 633 | wavelet-HHH | firstorder | 90Percentile |
| 634 | wavelet-HHH | firstorder | Minimum |
| 635 | wavelet-HHH | firstorder | Entropy |
| 636 | wavelet-HHH | firstorder | Range |
| 637 | wavelet-HHH | firstorder | Variance |
| 638 | wavelet-HHH | firstorder | 10Percentile |
| 639 | wavelet-HHH | firstorder | Kurtosis |
| 640 | wavelet-HHH | firstorder | Mean |
| 641 | wavelet-HHH | glrlm | ShortRunLowGrayLevelEmphasis |
| 642 | wavelet-HHH | glrlm | GrayLevelVariance |
| 643 | wavelet-HHH | glrlm | LowGrayLevelRunEmphasis |
| 644 | wavelet-HHH | glrlm | GrayLevelNonUniformityNormalized |
| 645 | wavelet-HHH | glrlm | RunVariance |
| 646 | wavelet-HHH | glrlm | GrayLevelNonUniformity |
| 647 | wavelet-HHH | glrlm | LongRunEmphasis |
| 648 | wavelet-HHH | glrlm | ShortRunHighGrayLevelEmphasis |
| 649 | wavelet-HHH | glrlm | RunLengthNonUniformity |
| 650 | wavelet-HHH | glrlm | ShortRunEmphasis |
| 651 | wavelet-HHH | glrlm | LongRunHighGrayLevelEmphasis |
| 652 | wavelet-HHH | glrlm | RunPercentage |
| 653 | wavelet-HHH | glrlm | LongRunLowGrayLevelEmphasis |
| 654 | wavelet-HHH | glrlm | RunEntropy |
| 655 | wavelet-HHH | glrlm | HighGrayLevelRunEmphasis |
| 656 | wavelet-HHH | glrlm | RunLengthNonUniformityNormalized |
| 657 | wavelet-HHH | glszm | GrayLevelVariance |
| 658 | wavelet-HHH | glszm | ZoneVariance |
| 659 | wavelet-HHH | glszm | GrayLevelNonUniformityNormalized |
| 660 | wavelet-HHH | glszm | SizeZoneNonUniformityNormalized |
| 661 | wavelet-HHH | glszm | SizeZoneNonUniformity |
| 662 | wavelet-HHH | glszm | GrayLevelNonUniformity |
| 663 | wavelet-HHH | glszm | LargeAreaEmphasis |
| 664 | wavelet-HHH | glszm | SmallAreaHighGrayLevelEmphasis |
| 665 | wavelet-HHH | glszm | ZonePercentage |
| 666 | wavelet-HHH | glszm | LargeAreaLowGrayLevelEmphasis |
| 667 | wavelet-HHH | glszm | LargeAreaHighGrayLevelEmphasis |
| 668 | wavelet-HHH | glszm | HighGrayLevelZoneEmphasis |
| 669 | wavelet-HHH | glszm | SmallAreaEmphasis |
| 670 | wavelet-HHH | glszm | LowGrayLevelZoneEmphasis |
| 671 | wavelet-HHH | glszm | ZoneEntropy |
| 672 | wavelet-HHH | glszm | SmallAreaLowGrayLevelEmphasis |
| 673 | wavelet-HHH | ngtdm | Coarseness |
| 674 | wavelet-HHH | ngtdm | Complexity |
| 675 | wavelet-HHH | ngtdm | Strength |
| 676 | wavelet-HHH | ngtdm | Contrast |
| 677 | wavelet-HHH | ngtdm | Busyness |
| 678 | wavelet-HHL | gldm | GrayLevelVariance |
| 679 | wavelet-HHL | gldm | HighGrayLevelEmphasis |
| 680 | wavelet-HHL | gldm | DependenceEntropy |
| 681 | wavelet-HHL | gldm | DependenceNonUniformity |
| 682 | wavelet-HHL | gldm | GrayLevelNonUniformity |
| 683 | wavelet-HHL | gldm | SmallDependenceEmphasis |
| 684 | wavelet-HHL | gldm | SmallDependenceHighGrayLevelEmphasis |
| 685 | wavelet-HHL | gldm | DependenceNonUniformityNormalized |
| 686 | wavelet-HHL | gldm | LargeDependenceEmphasis |
| 687 | wavelet-HHL | gldm | LargeDependenceLowGrayLevelEmphasis |
| 688 | wavelet-HHL | gldm | DependenceVariance |
| 689 | wavelet-HHL | gldm | LargeDependenceHighGrayLevelEmphasis |
| 690 | wavelet-HHL | gldm | SmallDependenceLowGrayLevelEmphasis |
| 691 | wavelet-HHL | gldm | LowGrayLevelEmphasis |
| 692 | wavelet-HHL | glcm | JointAverage |
| 693 | wavelet-HHL | glcm | SumAverage |
| 694 | wavelet-HHL | glcm | JointEntropy |
| 695 | wavelet-HHL | glcm | ClusterShade |
| 696 | wavelet-HHL | glcm | MaximumProbability |
| 697 | wavelet-HHL | glcm | Idmn |
| 698 | wavelet-HHL | glcm | JointEnergy |
| 699 | wavelet-HHL | glcm | Contrast |
| 700 | wavelet-HHL | glcm | DifferenceEntropy |
| 701 | wavelet-HHL | glcm | InverseVariance |
| 702 | wavelet-HHL | glcm | DifferenceVariance |
| 703 | wavelet-HHL | glcm | Idn |
| 704 | wavelet-HHL | glcm | Idm |
| 705 | wavelet-HHL | glcm | Correlation |
| 706 | wavelet-HHL | glcm | Autocorrelation |
| 707 | wavelet-HHL | glcm | SumEntropy |
| 708 | wavelet-HHL | glcm | MCC |
| 709 | wavelet-HHL | glcm | SumSquares |
| 710 | wavelet-HHL | glcm | ClusterProminence |
| 711 | wavelet-HHL | glcm | Imc2 |
| 712 | wavelet-HHL | glcm | Imc1 |
| 713 | wavelet-HHL | glcm | DifferenceAverage |
| 714 | wavelet-HHL | glcm | Id |
| 715 | wavelet-HHL | glcm | ClusterTendency |
| 716 | wavelet-HHL | firstorder | InterquartileRange |
| 717 | wavelet-HHL | firstorder | Skewness |
| 718 | wavelet-HHL | firstorder | Uniformity |
| 719 | wavelet-HHL | firstorder | Median |
| 720 | wavelet-HHL | firstorder | Energy |
| 721 | wavelet-HHL | firstorder | RobustMeanAbsoluteDeviation |
| 722 | wavelet-HHL | firstorder | MeanAbsoluteDeviation |
| 723 | wavelet-HHL | firstorder | TotalEnergy |
| 724 | wavelet-HHL | firstorder | Maximum |
| 725 | wavelet-HHL | firstorder | RootMeanSquared |
| 726 | wavelet-HHL | firstorder | 90Percentile |
| 727 | wavelet-HHL | firstorder | Minimum |
| 728 | wavelet-HHL | firstorder | Entropy |
| 729 | wavelet-HHL | firstorder | Range |
| 730 | wavelet-HHL | firstorder | Variance |
| 731 | wavelet-HHL | firstorder | 10Percentile |
| 732 | wavelet-HHL | firstorder | Kurtosis |
| 733 | wavelet-HHL | firstorder | Mean |
| 734 | wavelet-HHL | glrlm | ShortRunLowGrayLevelEmphasis |
| 735 | wavelet-HHL | glrlm | GrayLevelVariance |
| 736 | wavelet-HHL | glrlm | LowGrayLevelRunEmphasis |
| 737 | wavelet-HHL | glrlm | GrayLevelNonUniformityNormalized |
| 738 | wavelet-HHL | glrlm | RunVariance |
| 739 | wavelet-HHL | glrlm | GrayLevelNonUniformity |
| 740 | wavelet-HHL | glrlm | LongRunEmphasis |
| 741 | wavelet-HHL | glrlm | ShortRunHighGrayLevelEmphasis |
| 742 | wavelet-HHL | glrlm | RunLengthNonUniformity |
| 743 | wavelet-HHL | glrlm | ShortRunEmphasis |
| 744 | wavelet-HHL | glrlm | LongRunHighGrayLevelEmphasis |
| 745 | wavelet-HHL | glrlm | RunPercentage |
| 746 | wavelet-HHL | glrlm | LongRunLowGrayLevelEmphasis |
| 747 | wavelet-HHL | glrlm | RunEntropy |
| 748 | wavelet-HHL | glrlm | HighGrayLevelRunEmphasis |
| 749 | wavelet-HHL | glrlm | RunLengthNonUniformityNormalized |
| 750 | wavelet-HHL | glszm | GrayLevelVariance |
| 751 | wavelet-HHL | glszm | ZoneVariance |
| 752 | wavelet-HHL | glszm | GrayLevelNonUniformityNormalized |
| 753 | wavelet-HHL | glszm | SizeZoneNonUniformityNormalized |
| 754 | wavelet-HHL | glszm | SizeZoneNonUniformity |
| 755 | wavelet-HHL | glszm | GrayLevelNonUniformity |
| 756 | wavelet-HHL | glszm | LargeAreaEmphasis |
| 757 | wavelet-HHL | glszm | SmallAreaHighGrayLevelEmphasis |
| 758 | wavelet-HHL | glszm | ZonePercentage |
| 759 | wavelet-HHL | glszm | LargeAreaLowGrayLevelEmphasis |
| 760 | wavelet-HHL | glszm | LargeAreaHighGrayLevelEmphasis |
| 761 | wavelet-HHL | glszm | HighGrayLevelZoneEmphasis |
| 762 | wavelet-HHL | glszm | SmallAreaEmphasis |
| 763 | wavelet-HHL | glszm | LowGrayLevelZoneEmphasis |
| 764 | wavelet-HHL | glszm | ZoneEntropy |
| 765 | wavelet-HHL | glszm | SmallAreaLowGrayLevelEmphasis |
| 766 | wavelet-HHL | ngtdm | Coarseness |
| 767 | wavelet-HHL | ngtdm | Complexity |
| 768 | wavelet-HHL | ngtdm | Strength |
| 769 | wavelet-HHL | ngtdm | Contrast |
| 770 | wavelet-HHL | ngtdm | Busyness |
| 771 | wavelet-LLL | gldm | GrayLevelVariance |
| 772 | wavelet-LLL | gldm | HighGrayLevelEmphasis |
| 773 | wavelet-LLL | gldm | DependenceEntropy |
| 774 | wavelet-LLL | gldm | DependenceNonUniformity |
| 775 | wavelet-LLL | gldm | GrayLevelNonUniformity |
| 776 | wavelet-LLL | gldm | SmallDependenceEmphasis |
| 777 | wavelet-LLL | gldm | SmallDependenceHighGrayLevelEmphasis |
| 778 | wavelet-LLL | gldm | DependenceNonUniformityNormalized |
| 779 | wavelet-LLL | gldm | LargeDependenceEmphasis |
| 780 | wavelet-LLL | gldm | LargeDependenceLowGrayLevelEmphasis |
| 781 | wavelet-LLL | gldm | DependenceVariance |
| 782 | wavelet-LLL | gldm | LargeDependenceHighGrayLevelEmphasis |
| 783 | wavelet-LLL | gldm | SmallDependenceLowGrayLevelEmphasis |
| 784 | wavelet-LLL | gldm | LowGrayLevelEmphasis |
| 785 | wavelet-LLL | glcm | JointAverage |
| 786 | wavelet-LLL | glcm | SumAverage |
| 787 | wavelet-LLL | glcm | JointEntropy |
| 788 | wavelet-LLL | glcm | ClusterShade |
| 789 | wavelet-LLL | glcm | MaximumProbability |
| 790 | wavelet-LLL | glcm | Idmn |
| 791 | wavelet-LLL | glcm | JointEnergy |
| 792 | wavelet-LLL | glcm | Contrast |
| 793 | wavelet-LLL | glcm | DifferenceEntropy |
| 794 | wavelet-LLL | glcm | InverseVariance |
| 795 | wavelet-LLL | glcm | DifferenceVariance |
| 796 | wavelet-LLL | glcm | Idn |
| 797 | wavelet-LLL | glcm | Idm |
| 798 | wavelet-LLL | glcm | Correlation |
| 799 | wavelet-LLL | glcm | Autocorrelation |
| 800 | wavelet-LLL | glcm | SumEntropy |
| 801 | wavelet-LLL | glcm | MCC |
| 802 | wavelet-LLL | glcm | SumSquares |
| 803 | wavelet-LLL | glcm | ClusterProminence |
| 804 | wavelet-LLL | glcm | Imc2 |
| 805 | wavelet-LLL | glcm | Imc1 |
| 806 | wavelet-LLL | glcm | DifferenceAverage |
| 807 | wavelet-LLL | glcm | Id |
| 808 | wavelet-LLL | glcm | ClusterTendency |
| 809 | wavelet-LLL | firstorder | InterquartileRange |
| 810 | wavelet-LLL | firstorder | Skewness |
| 811 | wavelet-LLL | firstorder | Uniformity |
| 812 | wavelet-LLL | firstorder | Median |
| 813 | wavelet-LLL | firstorder | Energy |
| 814 | wavelet-LLL | firstorder | RobustMeanAbsoluteDeviation |
| 815 | wavelet-LLL | firstorder | MeanAbsoluteDeviation |
| 816 | wavelet-LLL | firstorder | TotalEnergy |
| 817 | wavelet-LLL | firstorder | Maximum |
| 818 | wavelet-LLL | firstorder | RootMeanSquared |
| 819 | wavelet-LLL | firstorder | 90Percentile |
| 820 | wavelet-LLL | firstorder | Minimum |
| 821 | wavelet-LLL | firstorder | Entropy |
| 822 | wavelet-LLL | firstorder | Range |
| 823 | wavelet-LLL | firstorder | Variance |
| 824 | wavelet-LLL | firstorder | 10Percentile |
| 825 | wavelet-LLL | firstorder | Kurtosis |
| 826 | wavelet-LLL | firstorder | Mean |
| 827 | wavelet-LLL | glrlm | ShortRunLowGrayLevelEmphasis |
| 828 | wavelet-LLL | glrlm | GrayLevelVariance |
| 829 | wavelet-LLL | glrlm | LowGrayLevelRunEmphasis |
| 830 | wavelet-LLL | glrlm | GrayLevelNonUniformityNormalized |
| 831 | wavelet-LLL | glrlm | RunVariance |
| 832 | wavelet-LLL | glrlm | GrayLevelNonUniformity |
| 833 | wavelet-LLL | glrlm | LongRunEmphasis |
| 834 | wavelet-LLL | glrlm | ShortRunHighGrayLevelEmphasis |
| 835 | wavelet-LLL | glrlm | RunLengthNonUniformity |
| 836 | wavelet-LLL | glrlm | ShortRunEmphasis |
| 837 | wavelet-LLL | glrlm | LongRunHighGrayLevelEmphasis |
| 838 | wavelet-LLL | glrlm | RunPercentage |
| 839 | wavelet-LLL | glrlm | LongRunLowGrayLevelEmphasis |
| 840 | wavelet-LLL | glrlm | RunEntropy |
| 841 | wavelet-LLL | glrlm | HighGrayLevelRunEmphasis |
| 842 | wavelet-LLL | glrlm | RunLengthNonUniformityNormalized |
| 843 | wavelet-LLL | glszm | GrayLevelVariance |
| 844 | wavelet-LLL | glszm | ZoneVariance |
| 845 | wavelet-LLL | glszm | GrayLevelNonUniformityNormalized |
| 846 | wavelet-LLL | glszm | SizeZoneNonUniformityNormalized |
| 847 | wavelet-LLL | glszm | SizeZoneNonUniformity |
| 848 | wavelet-LLL | glszm | GrayLevelNonUniformity |
| 849 | wavelet-LLL | glszm | LargeAreaEmphasis |
| 850 | wavelet-LLL | glszm | SmallAreaHighGrayLevelEmphasis |
| 851 | wavelet-LLL | glszm | ZonePercentage |
| 852 | wavelet-LLL | glszm | LargeAreaLowGrayLevelEmphasis |
| 853 | wavelet-LLL | glszm | LargeAreaHighGrayLevelEmphasis |
| 854 | wavelet-LLL | glszm | HighGrayLevelZoneEmphasis |
| 855 | wavelet-LLL | glszm | SmallAreaEmphasis |
| 856 | wavelet-LLL | glszm | LowGrayLevelZoneEmphasis |
| 857 | wavelet-LLL | glszm | ZoneEntropy |
| 858 | wavelet-LLL | glszm | SmallAreaLowGrayLevelEmphasis |
| 859 | wavelet-LLL | ngtdm | Coarseness |
| 860 | wavelet-LLL | ngtdm | Complexity |
| 861 | wavelet-LLL | ngtdm | Strength |
| 862 | wavelet-LLL | ngtdm | Contrast |
| 863 | wavelet-LLL | ngtdm | Busyness |

Abbreviations: glcm, gray-level co-occurrence matrix; glszm, gray-level size zone matrix; gldm, gray-level dependence matrix; glrlm, gray-level run length matrix; ngtdm, neighboring gray tone difference matrix.

**Table S3. The performance of models based on different number of selected features for pathological complete response prediction**

|  |  | **Training cohort (AUC)** | **Validation cohort (AUC)** |
| --- | --- | --- | --- |
| **Tumoral VOI** | T1+C-Top20 | 0.74 | 0.51 |
|  | T1+C-Top30 | 0.78 | 0.61 |
|  | T2WI-Top20 | 0.76 | 0.55 |
|  | T2WI-Top30 | 0.78 | 0.61 |
|  | DWI-ADC-Top20 | 0.84 | 0.55 |
|  | DWI-ADC-Top30 | 0.90 | 0.73 |
| **Peritumoral VOI** | T1+C-Top20 | 0.86 | 0.50 |
|  | T1+C-Top30 | 0.87 | 0.60 |
|  | T2WI-Top20 | 0.78 | 0.57 |
|  | T2WI-Top30 | 0.83 | 0.68 |
|  | DWI-ADC-Top20 | 0.87 | 0.55 |
|  | DWI-ADC-Top30 | 0.89 | 0.68 |

Abbreviations: AUC, area under the receiver operating characteristics curve; VOI, volume of interest; T1+C, contrast-enhanced T1-weighted imaging; T2WI, T2-weighted imaging; DWI-ADC, diffusion-weighted imaging quantitatively measured apparent diffusion coefficients.

**Table S4. Distribution of key radiomic features from tumoral and peritumoral volumes**

| **Radiomic features class** | **Tumoral VOI** | | | **Peritumoral VOI** | | |
| --- | --- | --- | --- | --- | --- | --- |
|  | T1+C | T2WI | DWI-ADC | T1+C | T2WI | DWI-ADC |
| **Shape** | 0 | 0 | 0 | 0 | 0 | 1 |
| **First-order** | 10 | 7 | 9 | 6 | 6 | 6 |
| **GLCM** | 6 | 11 | 9 | 9 | 9 | 9 |
| **GLSZM** | 3 | 5 | 6 | 3 | 5 | 4 |
| **GLDM** | 2 | 1 | 2 | 5 | 5 | 1 |
| **GLRLM** | 9 | 6 | 2 | 7 | 5 | 6 |
| **NGTDM** | 0 | 0 | 2 | 0 | 0 | 2 |

Abbreviations: VOI, volume of interest; T1+C, contrast-enhanced T1-weighted imaging; T2WI, T2-weighted imaging; DWI-ADC, diffusion-weighted imaging quantitatively measured the apparent diffusion coefficient; GLCM, gray-level co-occurrence matrix; GLSZM, gray-level size zone matrix; GLDM, gray-level dependence matrix; GLRLM, gray-level run length matrix; NGTDM, neighboring gray tone difference matrix.

**Table S5. The performance of each model for pathological complete response prediction.**

| Cohort | Model | Performance | | | | | |
| --- | --- | --- | --- | --- | --- | --- | --- |
|  |  | Sensitivity | Specificity | Accuracy | PPV | NPV | AUC (95%CI) |
| Training cohort | Tumor VOI radiomic model | 0.92 | 0.99 | 0.99 | 0.92 | 0.99 | 0.96 (0.91-1.00) |
|  | Tumor VOI DWI-ADC model | 0.80 | 0.98 | 0.97 | 0.77 | 0.98 | 0.90 (0.82-0.98) |
|  | Tumor VOI T1+C model | 0.53 | 0.94 | 0.90 | 0.50 | 0.94 | 0.78 (0.69-0.87) |
|  | Tumor VOI T2WI model | 0.58 | 0.94 | 0.90 | 0.54 | 0.95 | 0.78 (0.68-0.87) |
|  | Peri VOI radiomic model | 0.96 | 1.00 | 0.99 | 0.96 | 1.00 | 0.97 (0.92-1.00) |
|  | Peri VOI DWI-ADC model | 0.72 | 0.99 | 0.97 | 0.82 | 0.98 | 0.89 (0.82-0.96) |
|  | Peri VOI T1+C model | 0.76 | 0.95 | 0.93 | 0.64 | 0.97 | 0.87 (0.80-0.95) |
|  | Peri VOI T2WI model | 0.71 | 0.96 | 0.93 | 0.66 | 0.97 | 0.83 (0.74-0.92) |
|  | Tumor+Peri VOI radiomic model | 0.96 | 0.99 | 0.99 | 0.92 | 1.00 | 0.98 (0.93-1.00) |
|  | Tumor+Peri VOI DWI-ADC model | 0.94 | 0.98 | 0.97 | 0.78 | 0.99 | 0.92 (0.85-0.99) |
|  | Tumor+Peri VOI T1+C model | 0.79 | 0.97 | 0.95 | 0.73 | 0.98 | 0.88 (0.80-0.95) |
|  | Tumor+Peri VOI T2WI model | 0.68 | 0.99 | 0.96 | 0.87 | 0.96 | 0.88 (0.82-0.95) |
| Validation cohort | Tumor VOI radiomic model | 0.75 | 0.89 | 0.87 | 0.55 | 0.95 | 0.89 (0.78-1.00) |
|  | Tumor VOI DWI-ADC model | 0.67 | 0.90 | 0.86 | 0.55 | 0.94 | 0.73 (0.48-0.98) |
|  | Tumor VOI T1+C model | 0.92 | 0.46 | 0.52 | 0.21 | 0.97 | 0.61 (0.45-0.78) |
|  | Tumor VOI T2WI model | 1.00 | 0.29 | 0.39 | 0.18 | 1.00 | 0.61 (0.44-0.76) |
|  | Peri VOI radiomic model | 0.86 | 0.61 | 0.65 | 0.29 | 0.96 | 0.78 (0.62-0.94) |
|  | Peri VOI DWI-ADC model | 0.67 | 0.82 | 0.79 | 0.4 | 0.93 | 0.68 (0.44-0.92) |
|  | Peri VOI T1+C model | 0.91 | 0.38 | 0.45 | 0.19 | 0.97 | 0.60 (0.45-0.75) |
|  | Peri VOI T2WI model | 0.73 | 0.66 | 0.67 | 0.26 | 0.94 | 0.68 (0.54-0.83) |
|  | Tumor+Peri VOI radiomic model | 1.00 | 0.80 | 0.83 | 0.47 | 1.00 | 0.92 (0.85-1.00) |
|  | Tumor+Peri VOI DWI-ADC model | 0.67 | 0.82 | 0.79 | 0.40 | 0.93 | 0.66 (0.44-0.89) |
|  | Tumor+Peri VOI T1+C model | 1.00 | 0.34 | 0.43 | 0.19 | 1.00 | 0.68 (0.52-0.83) |
|  | Tumor+Peri VOI T2WI model | 0.82 | 0.50 | 0.54 | 0.21 | 0.94 | 0.65 (0.49-0.82) |

Abbreviations: PPV, positive predictive values; NPV, negative predictive values; CI, confidence interval; AUC, area under the receiver operating characteristics curve; VOI, volume of interest; Tumor VOI, tumoral volume of interest; Peri VOI, peritumoral volume of interest; Tumor+Peri VOI, tumoral and peritumoral volume of interest; T1+C, contrast-enhanced T1-weighted imaging; T2WI, T2-weighted imaging; DWI-ADC, diffusion-weighted imaging quantitatively measured apparent diffusion coefficients.

**Table S6. The performance for pathological complete response prediction of subgroup-analysis in the training cohort.**

| Subgroup | | pCR rate | Model performance | | | | | |
| --- | --- | --- | --- | --- | --- | --- | --- | --- |
|  |  |  | Sensitivity | Specificity | Accuracy | PPV | NPV | AUC (95%CI) |
| Molecular subtype |  |  |  |  |  |  |  |  |
|  | HR positive | 12/186 | 0.92 | 0.99 | 0.99 | 0.92 | 0.99 | 0.95 (0.85-1.00) |
|  | Her2 positive | 9/113 | 1.00 | 0.99 | 0.99 | 0.90 | 1.00 | 1.00 (0.99-1.00) |
|  | TNBC | 4/16 | 1.00 | 1.00 | 1.00 | 1.00 | 1.00 | 1.00 (1.00-1.00) |
| Clinical TNM stage |  |  |  |  |  |  |  |  |
|  | Stage I-II | 17/183 | 0.94 | 0.99 | 0.99 | 0.94 | 0.99 | 0.96 (0.89-1.00) |
|  | Stage III | 8/132 | 1.00 | 0.99 | 0.99 | 0.89 | 1.00 | 0.999 (0.996-1.00) |

Abbreviations: PPV, positive predictive values; NPV, negative predictive values; CI, confidence interval; AUC, area under the receiver operating characteristics curve; VOI, volume of interest; Tumor VOI, tumoral volume of interest; Peri VOI, peritumoral volume of interest; Tumor+Peri VOI, tumoral and peritumoral volume of interest; T1+C, contrast-enhanced T1-weighted imaging; T2WI, T2-weighted imaging; DWI-ADC, diffusion-weighted imaging quantitatively measured apparent diffusion coefficients.

**Table S7. The performance for pathological complete response prediction of subgroup-analysis in the validation cohort.**

| Subgroup | | pCR rate | Model performance | | | | | |
| --- | --- | --- | --- | --- | --- | --- | --- | --- |
|  |  |  | Sensitivity | Specificity | Accuracy | PPV | NPV | AUC (95%CI) |
| Molecular subtype |  |  |  |  |  |  |  |  |
|  | HR positive | 2/22 | 1.00 | 0.75 | 0.77 | 0.29 | 1.00 | 0.88 (0.61-1.00) |
|  | Her2 positive | 5/24 | 1.00 | 0.84 | 0.88 | 0.63 | 1.00 | 0.89 (0.76-1.00) |
|  | TNBC | 1/6 | 1.00 | 1.00 | 1.00 | 1.00 | 1.00 | 1.00 (NA-NA) |
| Clinical TNM stage |  |  |  |  |  |  |  |  |
|  | Stage I-II | 3/20 | 1.00 | 0.76 | 0.80 | 0.43 | 1.00 | 0.90 (0.74-1.00) |
|  | Stage III | 5/32 | 1.00 | 0.89 | 0.91 | 0.63 | 1.00 | 0.93 (0.83-1.00) |

Abbreviations: P PPV, positive predictive values; NPV, negative predictive values; CI, confidence interval; AUC, area under the receiver operating characteristics curve; VOI, volume of interest; Tumor VOI, tumoral volume of interest; Peri VOI, peritumoral volume of interest; Tumor+Peri VOI, tumoral and peritumoral volume of interest; T1+C, contrast-enhanced T1-weighted imaging; T2WI, T2-weighted imaging; DWI-ADC, diffusion-weighted imaging quantitatively measured apparent diffusion coefficients.

**Table S8. Multivariable analysis of pathological complete response status in relation to clinical characteristics**

| Characteristic | HR (95% CI) | p value |
| --- | --- | --- |
| Age, years |  | 0.599 |
| <35 vs ≥ 35 | 0.757 (0.267-2.142) | 0.599 |
| Number of tumors |  | 0.216 |
| 1 vs >1 | 0.453 (0.129-1.588) | 0.216 |
| Clinical TNM stage |  | 0.041 |
| I – II vs III | 0.433 (0.194-0.968) | 0.041 |
| Molecular subtypes |  | 0.001 |
| Luminal B vs Luminal A | 0.142 (0.016-1.301) | 0.084 |
| HER2-positive vs Luminal A | 0.177 (0.070-0.443) | <0.001 |
| Triple negative vs Luminal A | 0.557 (0.139-2.228) | 0.408 |

Abbreviations: TNM, tumor–node–metastasis; HER2, human epidermal growth factor receptors 2; ER, estrogen receptor; PR, progesterone receptors; Ki67, proliferation marker protein Ki-67.
